# Tolerance to heavy metals of bacteria from Brazilian deep aquatic environments

**DOI:** 10.1007/s00203-026-05226-5

**Published:** 2026-10-10

**Authors:** Agatha do Canto Shubeita, Caroline Menegotto-Silva, Rafela Wong Davi, Audrey Menegaz Proença, Cássio Stein Moura, Renata Medina-Silva

**Affiliations:** 1 Immunology and Microbiology Laboratory, School of Health and Life Sciences, Pontifical Catholic University of Rio Grande do Sul, PUCRS, Porto Alegre, Brazil; 2 Geobiology Group, School of Health and Life Sciences, Pontifical Catholic University of Rio Grande do Sul, PUCRS, Porto Alegre, Brazil; 3 Quantitative Evolutionary Ecological and Mechanistic Microbiology Group, Institut Cochin, Université Paris Cité, Paris, France; 4 Geofísica Stein, Porto Alegre, Brazil

**Keywords:** Aquatic bacteria, Biofilms, Guarani Aquifer, Heavy metals, Microbial tolerance, Pelotas Basin

## Abstract

Bacterial tolerance to heavy metals (HM) poses a significant risk to ecosystems and human health. This study evaluated tolerance to HM of 24 bacterial isolates from two deep aquatic environments in southern Brazil: the Guarani Aquifer System (GAS) and the Rio Grande Cone (RGC). GAS wells were in a region of intense agricultural activity, with water levels of iron, selenium and aluminum above regulatory limits. RGC is a deep-sea environment considered pristine, although presenting evidence of human impact. *Bacillus, Lysinibacillus, Pseudomonas* and *Paenibacillus* isolates were subjected to HM minimum inhibitory concentration tests, biofilm formation, scanning electron microscopy (SEM) and analysis of metal-related genes. 58% of GAS isolates were more tolerant to (FeSO_4_·7H_2_O), 75% to (NH_4_Al(SO_4_)_2_·12H_2_O), and 67% to (Na_2_SeO) than reference-comparative strains (RSs). RGC isolates were tolerant to ((CH_3_COO)_2_Hg) in low concentrations but equaled the RSs in (CuCl_2_·2H_2_O) and (NiCl_2_·6H_2_O) tolerance. Biofilm formation was intense in GAS isolates after 24 h-exposure to sodium selenite, while those from RGC showed weak biofilms under mercury (II) acetate. SEM revealed potential selenium nanoparticles and morphological alterations in GAS isolates, also evidencing external particles with high mercury content in marine bacteria. Three isolates from each environment presented genes related to selenium incorporation or mercury reduction. Results indicated that HM may interfere in structure and dynamics of microbial communities of these ecosystems, highlighting the relevance of these microbial phenotypic traits from unexplored (whereas impacted) aquatic environments as markers of water pollution and toxicity, and/or for bioremediation purposes, in line with the One Health principle.

**Supplementary Information:**

The online version contains supplementary material available at https://doi.org/10.1007/s00203-026-05226-5.

## Introduction

Aquatic environments are dominated by bacteria, archaea, protists, and fungi that drive essential biogeochemical processes, including organic matter degradation and nutrient cycling (Munn 2020). These microorganisms occur as planktonic or benthic, and frequently form biofilms, which are microbial aggregates embedded in an extracellular polymeric matrix that confers protection against environmental stress (Flemming et al. 2016; Flemming and Wuertz 2019).

Water bodies are naturally enriched with metals released through rock weathering and leaching (Guilherme et al. 2005; Maldonado, Wendling, 2009). Although many metals are essential micronutrients, when occurring in elevated concentrations—often resulting from agricultural, industrial, and urban inputs—they become toxic to microbial communities and consequently can interfere in biogeochemical cycling (Nogales et al. 2010; Steffen et al. 2015). Heavy metals are non-biodegradable and therefore persist for a long time in the environment (Loka et al. 1990). In this context, bacteria have evolved diverse tolerance mechanisms, such as efflux pumps, enzymatic detoxification, and biosorption. These tolerance traits are typically encoded by genes present in their chromosomes, plasmids or other mobile genetic elements. (Nies and Silver 1995; Vasudevan, 2001; Sykes 2009; Chatterjee et al. 2009; Jroundi et al. 2019). As these same mobile elements can harbor genes that confer resistance to different xenobiotics, metal stress can select for resistance to other biocides, like antibiotics and agrochemicals, raising concerns for both environment and human health (Di Cesare et al. 2016; Thomas et al. 2020). Therefore, metal tolerance can be considered a key phenotypic trait to be evaluated in microbial communities of natural environments, especially in those under urban, rural or industrial influence (Baker-Austin et al. 2006; Di Cesare et al. 2016; Thomas et al. 2020; Squadrone 2020).

Microbial habitats from deep subsurface and deep-sea environments are among the least explored on Earth, thus remaining largely uncharacterized regarding resistance and tolerance phenotypes in microbial communities. Nevertheless, these environments host metabolically active communities adapted to extreme pressure, temperature, and nutrient limitation (Hoehler and Jørgensen 2013). Moreover, although generally considered pristine environments, several studies indicate evidence they are disturbed by human activities, which include those that increase heavy metal concentrations (Williams et al. 2013; Gillard et al. 2019; Soares 2019; Jroundi et al. 2019; Moreno et al. 2021; Joshi et al. 2021). In this scenario, studies focused on analyzing the tolerance to heavy metals of bacteria from deep aquatic environments are still scarce. Moreover, ecosystems of the southern hemisphere are the least explored in such context. To contribute to the knowledge regarding this topic, this study focused on two deep aquatic environments in southern Brazil: the Guarani Aquifer System (GAS) and the Rio Grande Cone (RGC).

Although geographically and geologically distinct, GAS and RGC share some important ecological characteristics, such as isolation from direct atmospheric and anthropogenic contact, and low nutrient availability (Miller et al. 2015; Soares 2019; Moreno et al. 2021; Simão et al. 2026). Assessing metal tolerance in bacteria from both systems provides insights into the extent to which these relatively remote environments have been shaped by metal exposure. Anthropogenic inputs of metals have been introduced into the GAS through surface infiltrations (Foster et al. 2009; Terramoto et al. 2020; Moreno et al. 2021), and in RGC deep-sea sediments trough pollution from coastal areas (Zhuang and Gao 2014) that eventually reach oceanic sites, or even by navigation activities taken place in this area (da Silva et al. 2023). In the GAS, bacterial tolerance and resistance have been characterized in relation to agrochemicals and antibiotics, respectively (Silva et al. 2025), with no assessment of heavy metal tolerance. Likewise, resistance to antibiotics of bacterial isolates from the RGS sediment have been evaluated (Dias et al. 2026), but phenotypic and genetic characterization of metal tolerance across isolates from this site is still lacking**.**

Since it remains unclear how widespread metal tolerance is occurring among bacteria inhabiting these two deep aquatic ecosystems, the objective of this study was to phenotypically and genetically characterize bacteria isolated from GAS water samples and RGC deep marine sediments regarding their tolerance to heavy metals.

## Methods

### Origin of bacterial isolates

All the experimental procedures of the study (detailed below) are summarized in the schematic workflow of Supplementary Fig. S1.

Bacterial strains isolated from both selected sites, the Guarani Aquifer System (GAS) and the Rio Grande Cone (RGC), have been previously studied by our research group (Oliveira et al. 2021; Proença et al. 2022; Silva et al. 2025; Dias et al. 2026). The GAS is a confined groundwater system spanning approximately 1,100,000 km^2^ across the Paraná River Basin of Brazil, Paraguay, Uruguay, and Argentina, and is one of the largest freshwater reservoirs in South America (Foster et al. 2009). Anthropogenic inputs, including elevated concentrations of metals such as iron, aluminum and selenium, reach the aquifer through surface infiltration (Soares 2019; Moreno et al. 2021), and may already be shaping the resistance and tolerance profile of its native microbiota (Silva et al. 2025). The RGC, by contrast, is a deep-sea sedimentary feature covering roughly 250,000 km^2^ on the southern Brazilian continental margin (Pelotas Basin, Rio Grande do Sul), hosting a chemosynthesis-based community sustained by methane seepage (Miller et al. 2015; Giongo et al. 2016; ; Medina-Silva et al. 2018; Lopes Simão et al. 2026).

**Fig. 1:**
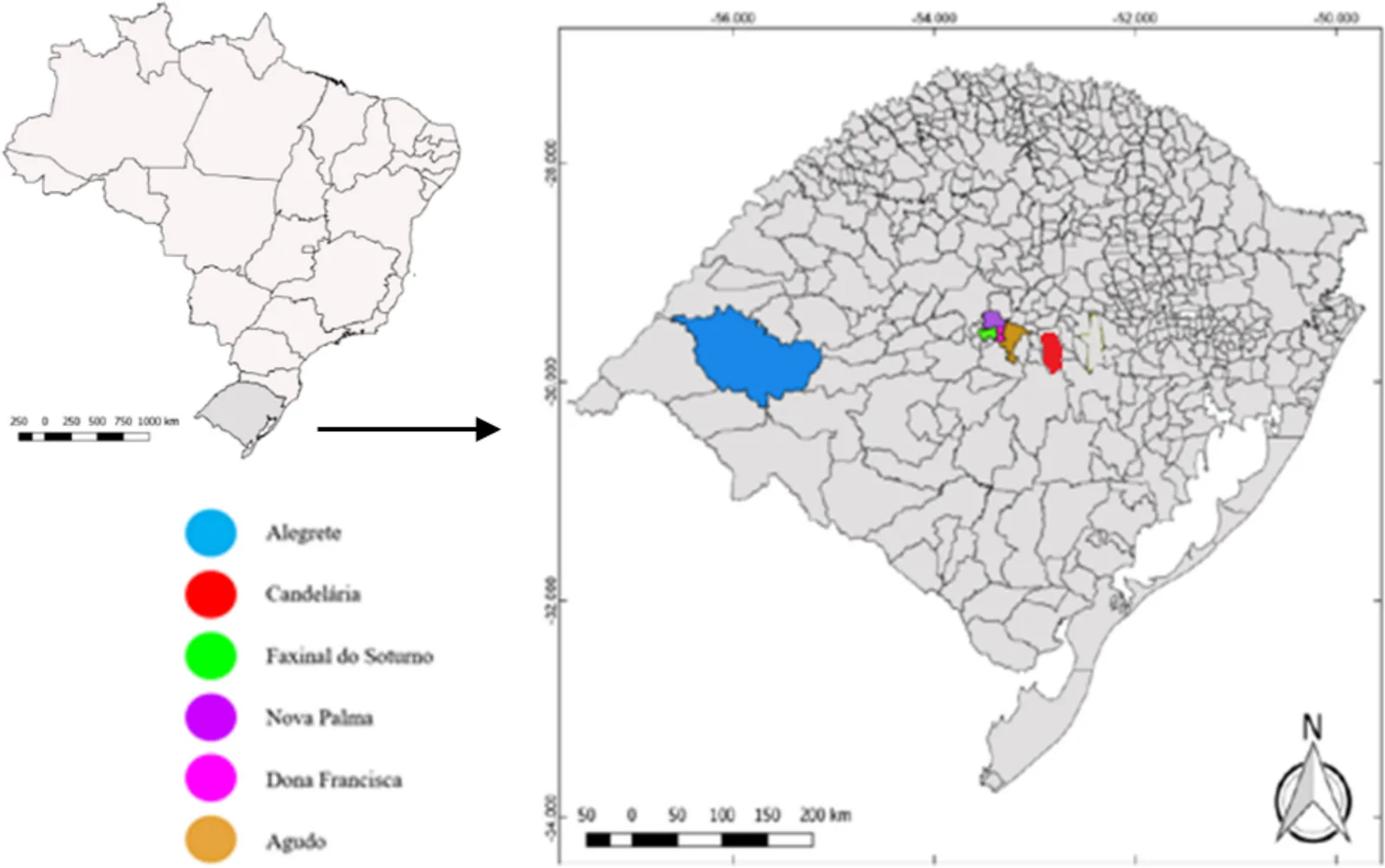
Location of the municipalities in which the water samples were collected from the Guarani Aquifer System (modified from Soares 2019)

Isolates from the GAS were previously obtained from water samples collected in 2018 from different wells that access this reservoir in Alegrete, Candelária, Faxinal do Soturno, Nova Palma, Dona Francisca and Agudo municipalities, all located in Rio Grande do Sul State, Brazil (Fig. 1) (Soares 2019). Water samples were collected aseptically and transported under refrigeration to the University. Bacterial isolates were then grown in Brain Heart Infusion (BHI) (pH 7.4 ± 0.2), at 25 °C for 24 h, purified and then stored at − 80 °C, in microtubes containing 1 mL of BHI broth with 30% glycerol (v/v) (Silva et al. 2025), until further use. Twelve GAS isolates were used in this research, from which nine were previously identified via 16S rRNA gene sequencing: one *Pseudomonas* (CAP2), one *Staphylococcus* (CAE1), three *Bacillus* (CAV19, CAV23 and VB5), and two *Lysinibacillus* (RF311 and GRU33), whereas two strains presented an unsupported identification (low bootstrap value in the phylogeny) (RF3121 and MEL13) as *Lysinibacillus*, and three presented no detected identification (unidentified isolates) (CAP10, CAP3 and NSP5) (Silva et al. 2025) (Table 1).

**Table 1:** Bacterial isolates from the Rio Grande Cone and the Guarani Aquifer System used in this study, their taxonomic identification (based in the 16S rRNA gene sequence), and collecting sites or regions, with corresponding site depths and coordinates (in UTM)

Rio Grande Cone*							Guarani Aquifer System**					
Isolates	Taxonomic identification	Site	Water column depth (m)	Sediment depth (cmbsf)	UTM x	UTM Y	Isolates	Taxonomic Identification	Region	Well depth (m)	UTM x	UTM Y
MD330.6	*Pseudomonas* sp.	PC 81	1369	50	614,622	6367.500	CAV19	*Bacillus* sp.	Alegrete	104	641,697	6,679,200
MD330.9	*Pseudomonas* sp.	PC 81	1369	50	614,622	6367.500	CAV23	*Bacillus* sp.	Alegrete	112	639,680	6,679,013
MD330.10	*Pseudomonas* sp.	PC 81	1369	100	614,622	6367.500	CAP2	*Pseudomonas* sp.	Alegrete	123	601,401	6,696,673
MD330.11	*Pseudomonas* sp.	PC 81	1369	100	614,622	6367.500	CAP10	Unidentified	Alegrete	123	601,401	6,696,673
MD332.6	*Pseudomonas* sp.	PC 81	1369	300	614,622	6367.500	CAP3	Unidentified	Alegrete	123	601,401	6,696,673
MD332.8	*Pseudomonas* sp.	PC 81	1369	300	614,622	6367.500	VB5	*Bacillus* sp.	Candelária	60	328,378	6,690,715
MET16	*Paenibacillus* sp.	PC 49	1200	100	609,316	6370,210	CAE1	*Staphylococcus* sp.	Nova Palma	140	277,021	6,737,934
MET17	*Paenibacillus* sp.	PC 49	1200	100	609,316	6370,210	RF3121	*Unsup. Lysinibacillus* sp.	Nova Palma	60	275,426	6,740,767
MR16	*Brevibacillus laterosporus*	PC 44	1311	50	608,815	6371,950	RF311	*Lysinibacillus* sp.	Nova Palma	60	275,426	6,740,767
MR06	*Bacillus megaterium*	PC 44	1311	50	608,815	6371,950	MEL13	*Unsup. Lysinibacillus* sp.	Fax Saturno	100	257,279	6,728,510
MR29	*Bacillus* sp.	PC 44	1311	50	608,815	6371,950	GRU33	*Lysinibacillus* sp.	Fax Saturno	40	253,331	6,731,263
MR38	*Bacillus* sp.	PC 44	1311	50	608,815	6371,950	NSP5	Unidentified	Agudo	> 20	289,075	6,736,722

*PC* piston core, *cmbsf* centimeters below sea floor, *Fax. Soturno* Faxinal do Soturno

^*^Source: Proença et al. (2022)

^**^Source: Silva e al. (2025)

**Fig. 2:**
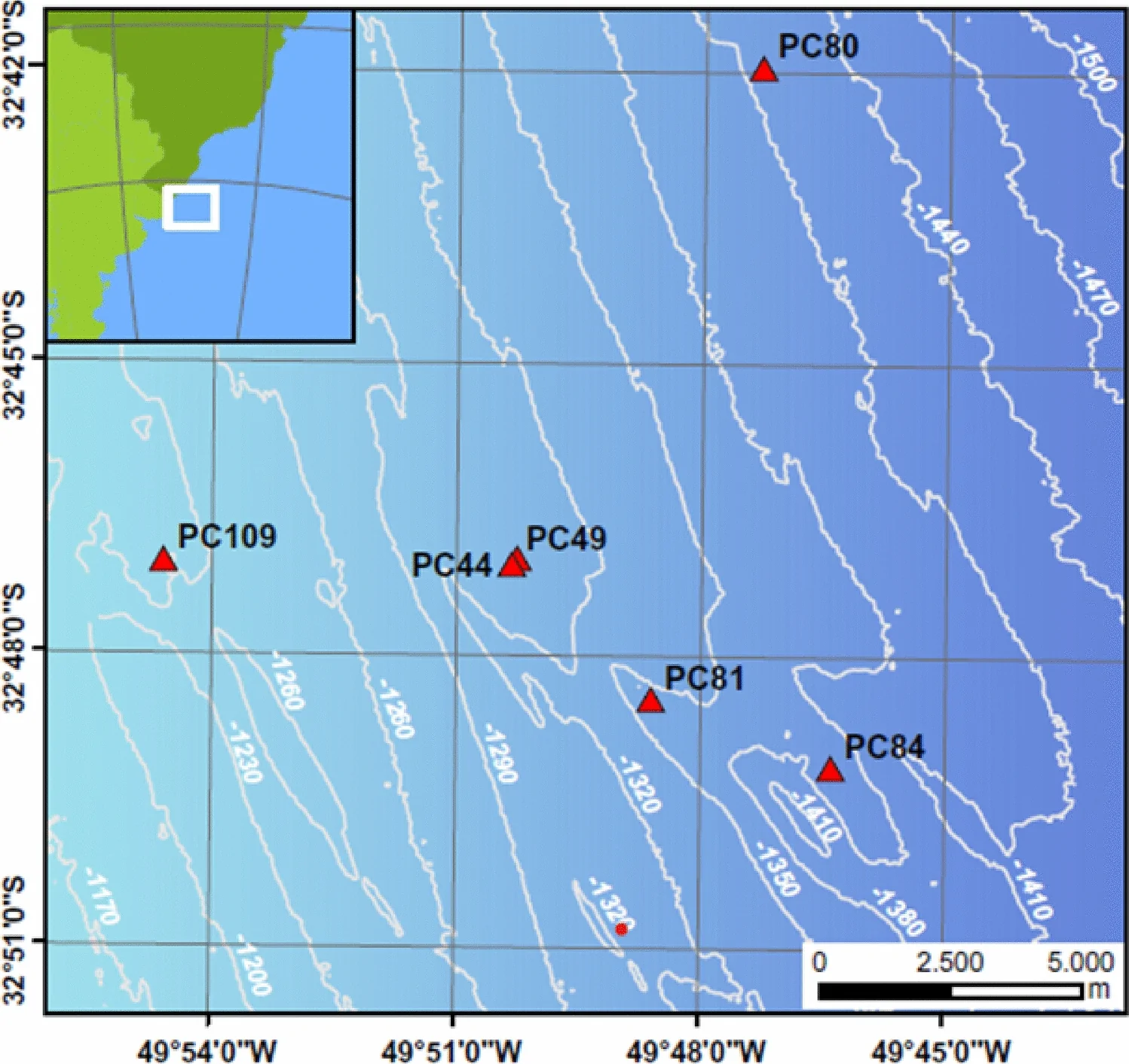
Geographic location of the five sites of Rio Grande Cone (Pelotas Basin) from which sediment samples were previously collected for microbial isolation. The isolates used in this study were obtained from samples of PC44, PC49 and PC81 sites (modified from Medina-Silva et al. 2018)

Bacterial isolates from the RGC, located in the Pelotas Basin, Brazil, were previously obtained (Proença et al. 2022) from sediment samples collected from 0 to 3 m below the seabed, during the MR11 (2011) and MD195 (2013) oceanographic missions, in five sites within a region of methane seepage, with a water column of about 1300 m, using a sediment piston core collector of approximately 60 m (Fig. 2). The isolates derived from the MR11 samples were obtained from 48 h cultures at 25 °C in BHI broth (pH 7.4 ± 0.2), or in YPD (1% yeast extract, 2% peptone and 2% dextrose) agar (pH 6.0), whereas MD195 bacteria were isolated in nitrate mineral salts (NMS) medium cultures (pH 7,2) for 48 h, at 30 °C or 37 °C, with methane being the only carbon source (Proença et al. 2022). Isolates were preserved at − 80 °C in microtubes containing 1 mL of BHI broth with glycerol or 5% dimethylsulfoxide (DMSO) in the proportion of 30% (v/v), until further use. Six isolates of *Pseudomonas* sp. (MD330.6, MD330.9, MD330.10, MD330.11, MD332.06 and MD332.08), three of *Bacillus* sp. (MR29, MR38, MR06), one isolate of *Brevibacillus* sp. (MR16) and two isolates of *Paenibacillus* sp. (MET16 and MET17) from three sites (PC44, PC49 and PC81) of the RGC were used in this research (Fig. 2, Table 1). All RGC *Pseudomonas* sp. isolates were previously described as resistant to aztreonam 30 µg (> 2048 µg/mL), and one isolate (MD330.09) to ceftadizyme 30 µg (128 µg/mL), whereas two *Paenibacillus* sp. (MET17 and MET16) were resistant to clindamycin 2 µg and erythromycin 15 µg (1 and 2 µg/mL, respectively) (Dias et al. 2026).

As total, 24 bacterial isolates (12 from each site) were recovered from stocks at − 80 °C by culturing in BHI broth, at 25 °C for 12–24 h. These cultures were plated by streaking on BHI agar and cultivated at 25 °C for 12–24 h, to obtain isolated colonies. The Gram staining was used to verify the purity of the isolates, which were analyzed under optical microscopy (1000×). In all tests, two reference strains were used to compare the results obtained for GAS and RGC results (as comparative strains): *P. aeruginosa* ATCC 27853 and *Bacillus cereus* ATCC 33019. The taxonomic identification of isolates from GAS and RGC was previously performed by Silva et al. (2025) and Proença et al. (2022), respectively. Their 16S rRNA sequences were then deposited in the National Center for Biotechnology Information (NCBI) database.

As mentioned above, bacterial isolates from the GAS have been previously described as tolerant to agrochemicals and resistant to antimicrobials (Silva et al. 2025). Bacterial strains isolated from RGC sediments have been shown to use diverse carbon sources, produce siderophores (Proença et al. 2022), and form biofilms on polyethylene microplastics (Oliveira et al. 2021). Antibiotic resistance was also described for bacterial isolates from the sediment of this deep-sea site (Dias et al. 2026). Moreover, heavy metal resistance genes (for mercury, zinc, and nickel) have been detected in the genome of the *Pseudomonas* sp. strain MD330.09 isolated from this site (Proença et al. 2022). The isolates of this study were selected based on this previous evidence of tolerance to herbicides (glyphosate and 2,4-D) and resistance to antibiotics of GAS isolates, or antibiotic resistance and heavy metal resistance genes detected in RGC bacteria. It is important to note that these criteria may have increased the likelihood of identifying metal tolerant strains, which may not reflect the overall prevalence of tolerance phenotypes in the broader GAS and RGC bacterial communities.

### Metal salts and media

GAS isolates were tested with iron (II) sulfate heptahydrate (FeSO_4_·7H_2_O), sodium selenite (Na_2_SeO_3_) and aluminum sulfate (NH_4_Al(SO_4_)_2_·12H_2_O), based on the findings of Moreno et al. (2021), that detected these metals in high concentrations in the same water GAS samples from which the isolates were cultured. These metal salts were tested at nominal concentrations of 1, 2, 4, 8, 16, 32, 64, 128 and 256 mM.

RGC isolates were tested with mercury (II) acetate ((CH_3_COO)_2_Hg), copper (II) chloride dihydrate (CuCl_2_·2H_2_O) and nickel (II) chloride hexahydrate (NiCl_2_·6H_2_O), chosen based on previous analysis of the genome of a *Pseudomonas* sp. MD330.06 (NCBI BioProject PRJNA616366), which indicated the presence of resistance genes to these metals, especially mercury (Proença et al. 2022). These metal salts were tested at nominal concentrations of 0.06, 0.125, 0.25, 0.5, 1, 2, 4, 8 and 16 mM.

Bacterial isolates were tested in minimum inhibitory concentration (MIC) experiments with Luria–Bertani (LB) agar medium plates (10 g/L tryptone, 5.0 g/L yeast extract, 5.0 g/L sodium chloride) supplemented individually with the distinct concentrations of metals salt employed. The LB agar medium was used as specified by the manufacturer (nominal pH 6.8–7.2 for LB agar).

### Minimum inhibitory concentration (MIC) tests

All marine and aquifer isolates were evaluated for tolerance to the metals described above using MIC tests, based on the protocol of Agarwal et al. (2020). Each isolate was grown on BHI agar for 24 h at 25 °C. A portion of isolated colonies was inoculated into BHI broth medium, and then incubated for 24 h at 25 °C. For the cell suspension, a volume of 1000 μl of the broth was centrifuged for 4 min at 4000 g, the supernatant was removed, the pellet was washed twice and resuspended with saline (0.85%), and the optical density at a wavelength of 600 nm (OD_600_) was adjusted to 0.1, using the Thermo Scientific Genesys 20 spectrophotometer.

A 96-well plate was used for serial dilutions of the cell suspensions of each isolate. Firstly, 270 μl of saline (0.85%) was added to each microwell. To prepare the first dilution (10^−1^), 30 μl of cell suspension was inoculated in the first microwell and then diluted up to 10^−7^. Subsequently, a 2.5 μl drop of each dilution was seeded on LB agar plates prepared, in duplicates, with different concentrations of the metals salts: 1, 2, 4, 8, 16, 32, 64, 128 and 256 mM for sodium selenite; 1, 2, 4, 8 and 16 mM for aluminum sulfate and iron (II) sulfate; 0.06, 0.125, 0.25, 0.5, 1, 2, 4 for mercury (II) acetate; and 1, 2, 4, 8 and 16 mM for copper (II) chloride and nickel (II) chloride. Moreover, two LB-agar plates with no metals were also included, to serve as negative controls. Plates were all incubated for 24–48 h at 25 °C. After this period, bacterial growth was observed and recorded for each isolate (Agarwal et al. 2020). The lowest concentration of metal that inhibited the appearance of colonies was considered the MIC for each isolate.

### Influence of metals on biofilm formation

These assays were performed according to Barros et al. (2014), exposing GAS isolates to sodium selenite and RGC isolates to mercury (II) acetate. For cell suspension, the isolates were cultivated in BHI broth at 25 °C until exponential phase (OD_600_ of 0.8). Then, they were centrifuged for 4 min at 4000 g, washed twice, and resuspended with saline solution (0.85%), for an OD_600_ of 0.8, using the Thermo Scientific Genesys 20 spectrophotometer. A 96-well polystyrene plate was used for biofilm formation, to which 100 μl of 10× or 100× of metal salts solutions were diluted in the BHI broth and a 1 μl aliquot of cell suspensions was added in each well. The concentrations of metal salts employed for biofilms treatments were defined according to MICs values obtained for isolates, within the distribution range of MICs observed for sodium selenite or mercury (II) acetate. GAS isolates that exhibited selenite MIC values of 1, 2, 4, 8, 16, 32 and 64 mM had biofilms exposed to these same concentrations, whereas those with MICs of 128 and 256 mM were exposed to 2, 4, 8, 16, 32, 64, 128 and 256 mM of this salt. Likewise, isolates from RGC with MIC value of 0.06 mM for mercury (II) had biofilms treated with 0.06, 0.125, 0.25, 0.5 and 1 mM, and those with MIC of 0.125 mM were exposed to 0.06, 0.125, 0.25, 0.5, 1, 2, 4, 8 and 16 mM of this metal. Negative controls were prepared solely with BHI broth, and the sample 0 mM was prepared only with BHI broth with cell suspension. The plates were incubated for 24 and 48 h at 25 °C. The supernatant was removed and the microwells were washed twice with PBS to remove non-adherent cells. In each microwell, 200 μl of 0.25% crystal violet was added, and the plate was incubated for 30 min at 25 °C. The crystal violet was removed and the microwells were washed twice with PBS. The crystal violet absorbed by biofilms was solubilized with 95% ethanol, and the plate was incubated again for 30 min at 25 °C. The crystal violet absorbance was then read at 595 nm on the SpectraMax^®^ 190 Microplate Reader (Molecular Devices) (Chari et al. 2014, modified). All tests were conducted in triplicate.

The absorbance results were averaged, and the biofilms were classified according to Stepanović et al. (2004): the cutoff OD (ODc) was defined as three standard deviations above the mean OD of the negative control. An OD ≤ ODc = no biofilm producer, ODc < OD ≤ (2 × ODc) = weak biofilm producer, (2 × ODc) < OD ≤ (4 × ODc) = moderate biofilm and (4 × ODc) < OD = strong biofilm producer.

### Scanning electron microscopy (SEM)

Two isolates belonging to different genera from each environments (GAS and RGC) that presented a high tolerance pattern to the tested metals were submitted to SEM analysis to investigate the potential presence of elemental selenium nanoparticles (SeNPS) in isolates grown in sodium selenite, and possible cell deformities in isolates grown in mercury (II) acetate (as observed by Oremland et al. 2004; Shakibaie et al. 2013; Jia et al. 2014; Joshi et al. 2021; ). Cell suspensions were prepared with a portion of isolates’ colonies grown in LB-agar supplemented with mercury (II) acetate (0.06 mM) or selenite sodium (128 mM)—prepared as described above—and then fixed with 25% glutaraldehyde for at least 5 days. Then, cell suspensions were centrifuged for 4 min at 4000 g, the pellet was washed with 0.2 M phosphate buffer and subjected to dehydration with increasing acetone concentrations (30%, 50% and 70%). Samples were spread in glass overslips, air dried and metallized with gold (BAL-TEC– SCD 050—Sputter Coater, Balzers, Liechtenstein). A secondary electron detector at 20.0 kV (Scanning Electron Microscopy, INSPECT-F50, FEI Company Inspect, Eindhoven, Netherlands) was used to examine and obtain the images. For the elemental analysis of the samples, a Field Emission Scanning Electron Microscope (INSPECT-F50, FEI Company) was used to perform an Energy Dispersive X-ray spectrometry (EDS). To distinguish elemental information of samples from background signals (to validate the elemental findings), measures of EDS only from the glass coverslip were performed for comparison with those of bacterial cells. Moreover, all structures and cells detected during SEM analysis were measured and compared. These analyses were performed by the Central Laboratory of Microscopy and Microanalysis (LabCEMM) from Pontifical Catholic University of Rio Grande do Sul (PUCRS).

### Detection of mercury resistance and selenium metabolism genes by PCR

The DNA of isolates was extracted as described by Rademaker (1997), using the guanidine thiocyanate-EDTA-Sarkosyl (GES) solution. The presence of two genes was investigated by PCR in GAS and RGC isolates that presented high MIC values for sodium selenite and mercury (II) acetate, respectively. For GAS isolates, the gene *selD* (490–510 bp), coding for the SelD protein, which is related with the ability of Se incorporation into selenoproteins, was selected. Moreover, *selD* gene is considered as a common signature for bacterial Se utilization, differently from other Se utilization traits, which present unique genes depending on the bacterial group (Romero et al. 2005; Zhang et al. 2006). In RGC isolates, we investigated the mercury-resistance marker gene *merA* (fragments of 431–443 bp), which encodes the mercuric reductase enzyme MerA, and has been previously detected in genomic sequences of *Pseudomonas* isolate MD330.6 (Proença et al. 2022). So, this PCR screening for *merA* and *selD* genes was performed as a targeted assessment of two well-characterized and widespread bacterial metabolic pathways relevant to mercury (Rasmussen et al. 2008; Sotero-Mertins et al. 2008) and selenium (Romero et al. 2005; Zhang et al. 2006), respectively.

The gene *selD* was searched in VB5, GRU33, NSP5, MEL13 and CAP3 GAS isolates. The sequence of *selD* primers was: *selDf* (5′-GCAAAAATTGAATCCCGTGT-3′) and *selDr* (50 TGTTGTTCTTTTGGCAGAACC-30) (Estrada et al. 2020). The amplification reactions were performed in 20 μl PCR reaction mixture, with 1.87 mM MgCl_2_, 1.25 U of DNA polymerase, 100 mM of dNTPs and 1 X Taq buffer and 1.5 μM of each primer. The PCR conditions were initial denaturation at 94 °C for 5 min, 30 cycles of 94 °C for 1 min, 58 °C for 1 min and 72 °C for 1 min, final extension of 72 °C for 5 min (Estrada et al. 2020). PCR for the *merA* gene was carried out in MD330.06, MD330.09, MD330.11, MD332.08 and MET16, MR06, MR16, MR29 and MR38 RGC isolates, with the primers set *F1merA* 5′-TCGTGATGTTCGACCGCT-3′ and *F2merA* 5′-TACTCCCGCCGTTTCCAAT-3′ (Sotero-Martins et al. 2008). The amplification reactions were performed in 25 μl PCR reaction mixture, containing 1 U μl-1 Taq polymerase, 1 X enzyme buffer, 100 μM of each deoxynucleoside triphosphate (dNTPs), 1 mM MgCl_2_ and 0.96 μM of each primer. The PCR conditions were pre-denaturation step at 94 °C for 5 min followed by 30 cycles of 94 °C for 1 min, 52 °C for 1 min, an extension step at 72 °C for 1 min and final extension at 72 °C for 7 min (Joshi et al. 2021). All the amplification results were verified by electrophoresis 1.5% gel agarose stained with ethidium bromide.

Moreover, in a previous study we identified the *mer* operon in *Pseudomonas* isolate MD330.06 through whole genome sequencing (NCBI BioProject PRJNA616366, Proença et al. 2022). Thus, this strain carries the *merA* gene (and possibly the corresponding MerA enzyme), which is annotated in its contig c41 (GenBank: JABULD010000718.1, locus_tag = HT737_28405). Thus, using ClustalW 2.0 (Larkin et al. 2007) we performed an alignment search between the contig nucleotide sequence and the *merA* primers used, which were developed based on the *merA* gene of *Citrobacter freundii* (GenBank AJ418049.1). A second alignment between *merA* gene from *Pseudomonas* MD330.6 and the *Citrobacter freundii* reference strain was also performed.

### Data compilation

The isolates that revealed to present distinguished properties regarding metal tolerance were compiled according to their taxa and origin (Rio Grande Cone or Guarani Aquifer System). The properties considered for this selection were: high values for metals’ MICs, ability to form biofilms under high concentrations of selenite or mercury (II), presence of genes involved in tolerance to metals, and/or observations in SEM images that may be related to metals’ tolerance.

## Results

### Minimum inhibitory concentration (MIC) assays

The results obtained from MIC assays performed with the 12 GAS isolates are reported in Table 2. For the purposes of this study, MIC values were classified as high or low relative to those obtained for the reference-comparative strains (RSs) ATCC 27853 and ATCC 33019. The results indicated distinct tolerance patterns among isolates for each metal tested. For iron (II) sulfate, MICs were identical or inferior to those obtained for both RSs, ranging between 8 mM (42% of isolates) and 16 mM (58% of isolates). For aluminum sulfate, MIC values were mostly 8 mM (75% of isolates), which was above the MIC detected in ATCC 27853, but the same observed in ATCC 33019. In contrast, for sodium selenite, most GAS isolates presented MIC values that were two or four times those detected in the RSs. Moreover, isolates submitted to sodium selenite presented colonies with a distinct orange-red color. These results also highlighted *Bacillus* sp. VB5 as the only GAS isolate in which all three metals showed their highest observed MICs, indicating a distinguished tolerance to iron (II) sulfate, aluminum sulfate and sodium selenite. Additionally, the unidentified isolates CAP3 and NSP5 also exhibited tolerance to high concentrations of the three metals tested.

**Table 2:** Minimum inhibitory concentrations (MIC) of iron (II) sulfate (FeSO_4_·7H_2_O), selenium (Na_2_SeO_3_) and aluminum sulfate (NH_4_Al(SO_4_)_2_·12 H_2_O) for the 12 bacterial isolates from the Guarani Aquifer System and for the reference-comparative strains (ATCC 27853 and ATCC 33019)

Isolates	Taxonomic identification	Minimum inhibitory concentrations (mM)		
		Iron (II) sulfate	Aluminum sulfate	Sodium selenite
CAP2	*Pseudomonas* sp.	16	8	16
CAP10	Unidentified	16	8	4
CAP3	Unidentified	16	8	128
CAE1	*Staphylococcus* sp.	8	8	32
CAV19	*Bacillus* sp.	16	8	64
CAV23	*Bacillus* sp.	8	8	64
GRU33	*Lysinibacillus* sp.	8	4	256
VB5	*Bacillus* sp.	16	8	256
RF3121	*Lysinibacillus* sp.	16	4	8
RF311	Unsup*. Lysinibacillus*	8	8	64
MEL13	Unsup. *Lysinibacillus*	8	4	128
NSP5	Unidentified	16	8	128
ATCC 27853	*P. aeruginosa*	16	4	64
ATCC 33019	*B. cereus*	16	8	64

Isolates with distinguished results are highlighted: the isolate that presented the highest MIC values of all metals is dark grey, and those with the highest iron (II) sulfate and aluminum sulfate MICs and the second highest selenium MIC are in light gray

Regarding the RGC bacteria (Table 3), mercury (II) acetate MIC for all *Bacillus* and *Paenibacillus* isolates was 0.125 mM, equal to that of the RSs, whereas most *Pseudomonas* sp. (4 out of 6 isolates) exhibited a lower MIC of 0.06 mM. For copper (II) chloride, most isolates showed MICs equal to (67% of isolates) or below (25%) that of the RSs (8 mM). Differently, *Paenibacillus* sp. MET17 presented the highest MIC value for copper (II) (16 mM). Similarly, nickel (II) chloride MICs of most isolates were the same (50%) or below (33%) that of the RSs (8 mM), while two isolates reached 16 mM. A few isolates showed remarkable tolerance levels: *Pseudomonas* sp. MD332.08 and *B. megaterium* MR06 reached high mercury (II) acetate MICs and the highest nickel (II) chloride MIC observed. This *Pseudomonas* sp. strain also exhibited the second highest copper (II) chloride MIC, behind only the *Paenibacillus* sp. MET17.

**Table 3:** Minimum inhibitory concentrations (MIC) of mercury ((CH_3_COO)2Hg), copper (II) chloride (CuCl_2_·2H_2_O) and nickel (II) chloride (NiCl_2_·6H_2_O) for the 12 bacterial isolates from the Rio Grande Cone, and for the reference-comparative strains (ATCC 27853 and ATCC 33019)

Isolates	Taxonomic identification	Minimum inhibitory concentrations (mM)		
		Mercury (II) acetate	Copper (II) chloride	Nickel (II) chloride
MD330.6	*Pseudomonas* sp.	0.06	8	8
MD330.9	*Pseudomonas* sp.	0.06	8	4
MD330.10	*Pseudomonas* sp.	0.06	8	8
MD330.11	*Pseudomonas* sp.	0.125	8	8
MD332.6	*Pseudomonas* sp.	0.06	8	8
MD332.8	*Pseudomonas* sp.	0.125	8	16
MET17	*Paenibacillus* sp.	0.06	16	8
MET16	*Paenibacillus* sp.	0.125	8	8
MR16	*B. laterosporus*	0.125	8	4
MR06	*B. megaterium*	0.125	2	16
MR29	*Bacillus* sp.	0.125	4	4
MR38	*Bacillus* sp.	0.125	4	4
ATCC 27853	*P. aeruginosa*	0.125	8	8
ATCC 33019	*B. cereus*	0.125	8	8

Isolates with distinguished results are highlighted: the isolate that presented the highest MIC values of mercury and nickel (II) chloride, and the second highest of copper (II) chloride is dark grey, whereas those that exhibited the highest MIC of at least one metal are in light gray

### Influence of metals on biofilm formation

**Fig. 3:**
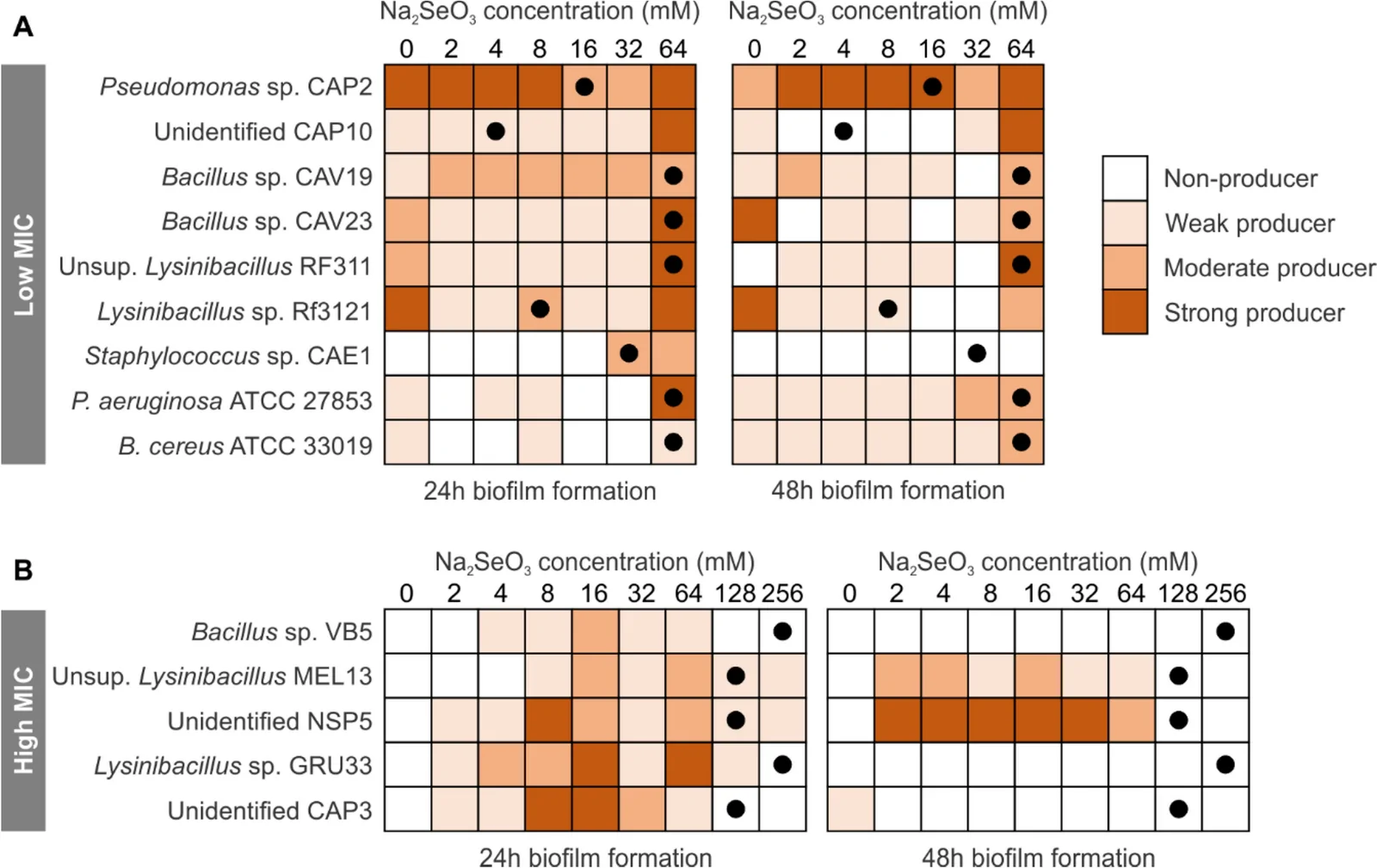
Ability of biofilm formation under the influence of Na_2_SeO_3_ of 12 bacterial isolates from the Guarani Aquifer System and of reference-comparative strains (ATCC 27853 and ATCC 33019), in 24 and 48 h. The sodium selenite MIC values obtained for each isolate (from Table 2) are indicated in black dots. **A** Isolates on which sodium selenite presented MIC values up to 64 mM; **B** Isolates on which sodium selenite MIC values were higher than 64 mM. The different colors indicate the intensity of the biofilm formation, as labeled in the color box

**Fig. 4:**
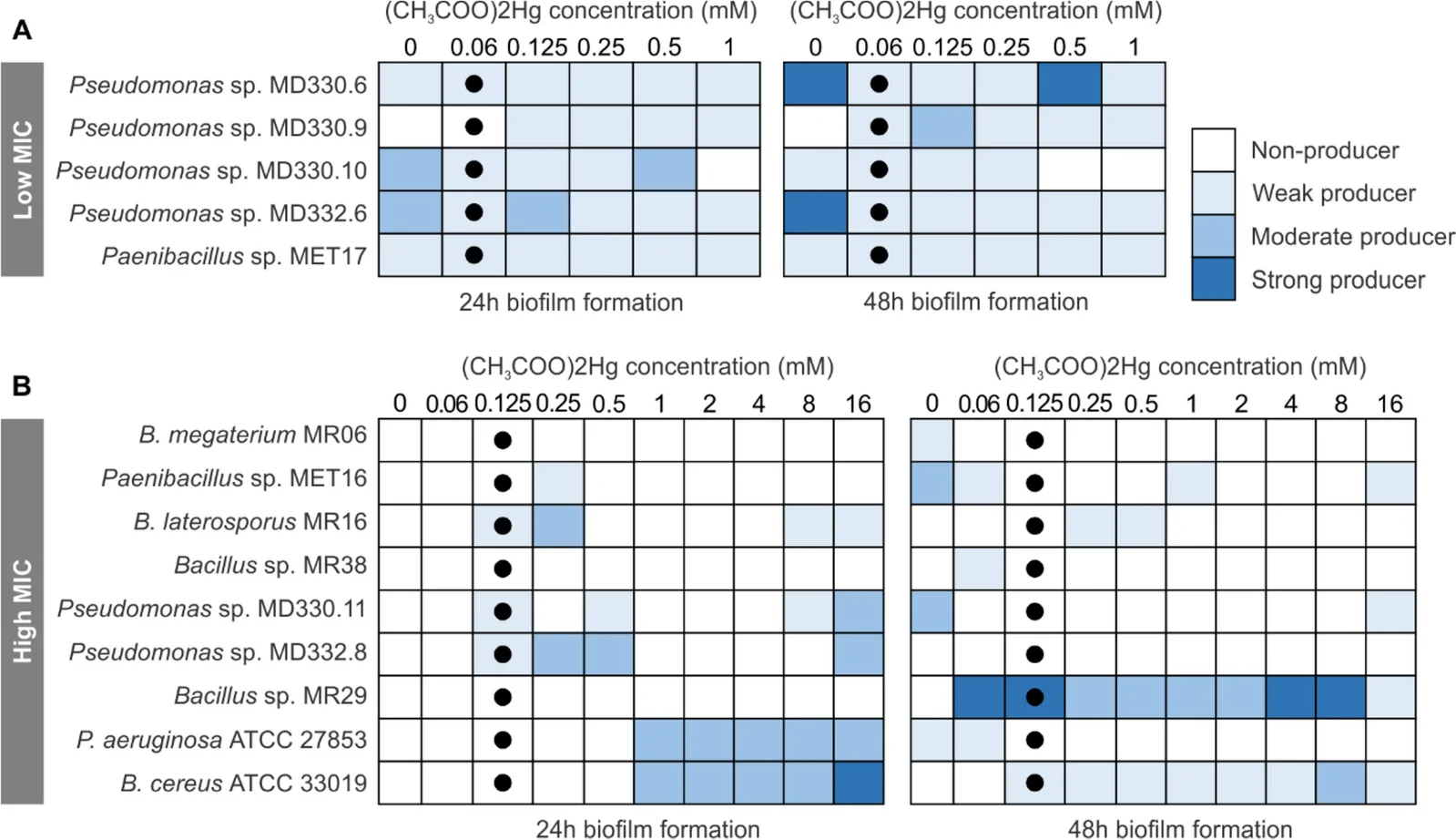
Ability of biofilm formation under the influence of (CH_3_COO)2Hg of 12 bacterial isolates from the Rio Grande Cone and of reference-comparative strains (ATCC 27853 and ATCC 33019), in 24 and 48 h. **A** isolates on which mercury (II) acetate MIC values were 0.06 mM; **B** isolates on which mercury (II) acetate MIC values were 0.125 mM. The different colors indicate the intensity of the biofilm formation, as labeled in the color box. The mercury (II) acetate MIC values obtained for each isolate (from Table 3) are indicated in black dots

All isolates from the GAS and RGC were tested for their ability to form biofilms under the influence of sodium selenite and mercury (II) acetate (Figs. 3, 4, respectively), for 24 and 48 h, including concentrations exceeding their MIC values.

Among GAS isolates in which sodium selenite presented MIC values equal or lower than 64 mM, control biofilm formation was variable, ranging from absent (CAE1) or weak (CAP10, CAV19), to moderate or strong (CAV23, RF3121, CAP2) (Fig. 3A). Under 24-h sodium selenite exposure, all these isolates were able to form biofilms in at least some concentrations. Biofilm production became more heterogeneous at 48 h, with some isolates (RF, CAV and CAE1) decreasing their biofilm-forming ability at some concentrations. Differently, RF311 formed biofilms only in the presence of sodium selenite. Despite this variability, all isolates demonstrated moderate or strong biofilm formation at 24 h under 64 mM of this salt, which is equal to or higher than their corresponding MICs (Table 2). CAP isolates were particularly notable for producing biofilms above their respective MICs, though with contrasting intensities between isolates (CAP10 remaining weak, CAP2 reaching strong formation up to 64 mM). In contrast, ATCC strains showed mainly weak or no biofilm formation at 24 h, and weak or moderate biofilms at 48 h (Fig. 3A).

For GAS isolates on which sodium selenite MICs exceeded 64 mM (Fig. 3B), no control biofilms were observed at 24 h. Biofilm formation and intensity by NSP5, CAP3 and GRU33 increased with metal concentration up to an intermediate range (2–16 mM), in which they reached moderate to strong biofilms. At higher concentrations (128–256 mM) most isolates (VB5, MEL13, NSP5, GRU33, CAP3) showed weak or no biofilm formation at 24 h. At 48 h, this metal-dependent pattern persisted: the two isolates with the highest sodium selenite MICs (GRU33 and VB5) formed no biofilms in controls, while MEL13 and NSP5 formed biofilms only under sodium selenite exposure, with NSP5 notably sustaining strong biofilm formation across a broad range (2–32 mM) before declining at 64 mM and ceasing at 128 mM (Fig. 3B).

Among RGC isolates with a mercury (II) acetate MIC of 0.06 mM (Fig. 4A), most maintained weak biofilms along treatments, with some increasing intensity at 48 h. Moreover, they were all able to produce biofilms under mercury (II) acetate concentrations exceeding their MICs (in most cases, by over 16×), with varied individual responses. MET17 showed constant, albeit weak, biofilm formation across all concentrations and both time points, while MD330.10 formed mostly weak biofilms up to 0.5 mM (24 h) and 0.25 mM (48 h). Differently, MD330.9 did not produce biofilms in the absence of mercury (II) acetate but presented weak or moderate formation under exposure up to 1 mM. MD330.06 and MD332.06 also formed biofilms under the highest concentration tested at both time points, with MD330.06 notably producing a strong biofilm at 0.5 mM, indicating a stronger biofilm-promoting response to this metal compared to the other isolates in this group (Fig. 4A).

Regarding RGC isolates with a mercury (II) acetate MIC of 125 mM (Fig. 4B), biofilm formation was generally weaker than that observed for isolates with lower MICs. At 24 h, none exhibited biofilm formation below 0.06 mM. MR16, MD330.11 (both *Pseudomonas* sp.) and MD332.08 (*B. laterosporus*) began forming biofilms at 0.125 mM, reaching moderate intensity at 16 mM. The RSs ATCC 27853 and ATCC 33019 formed moderate to strong biofilms from 1 to 16 mM. At 48 h, responses became more heterogeneous, with MR06, MET16, MD330.11 and ATCC 27853 forming weak to moderate control biofilms. Under treatment, MD330.11 and MET16 formed only weak biofilms at some concentrations (including 16 mM), MD332.08 unexpectedly formed none, and MR16 formed weak biofilms only at 0.25–0.5 mM. *Bacillus* MR29 was distinguished, as it formed strong or moderate biofilms from 0.06 to 8 mM, weakening only at 16 mM. ATCC 33019 formed weak biofilms from 0.125 to 16 mM and ATCC 27853 did it only up to 0.06 mM.

### Scanning electronic microscopy (SEM)

**Fig. 5:**
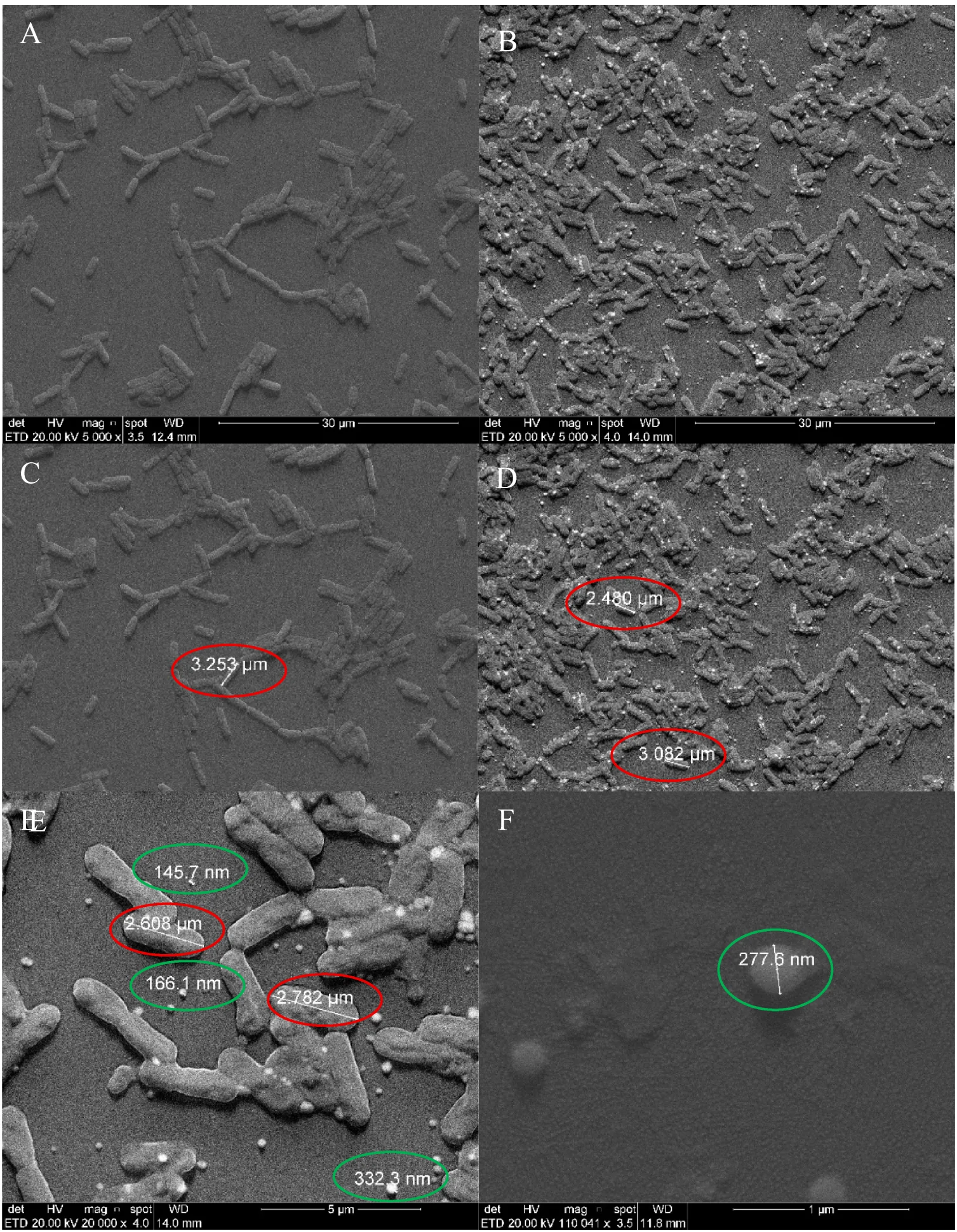
Scanning electron microscopy (SEM) analysis of GAS isolate VB5. VB5 cultures grown for 24 h at 25 °C without sodium selenite (**A**, **C**) and with 128 mM sodium selenite (**B**, **D**, **E**, **F**). VB5 cell measures are presented in **C**, **D** and **E** (red). Nanoparticles found in the sodium selenite treatment measures are indicated in **E** and **F** (green). Magnification of 5000× in **A**–**D**, 20,000× in **E** and 110,000× in **F**

**Fig. 6:**
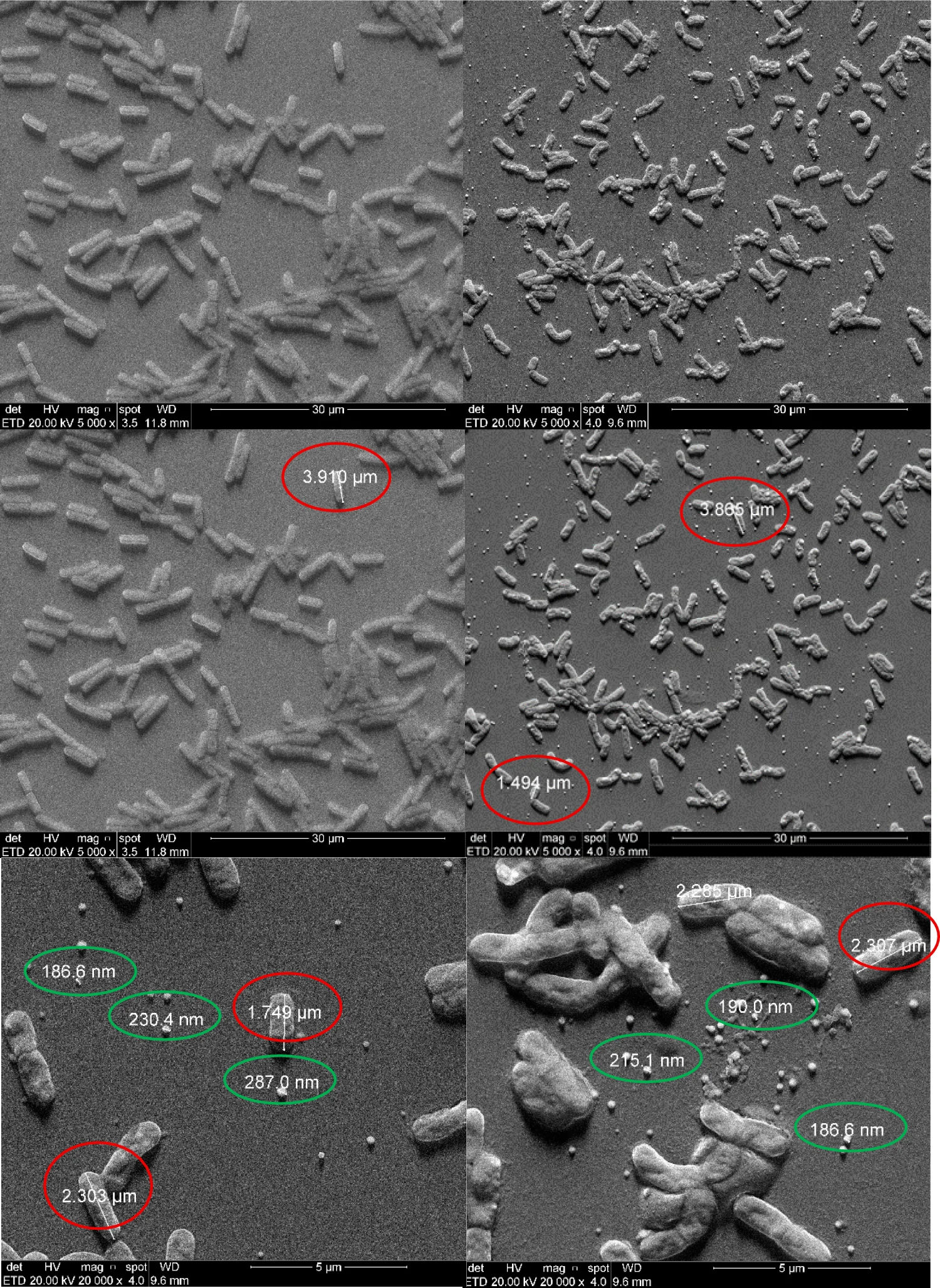
Scanning electron microscopy (SEM) analysis of GAS isolate GRU33. GRU33 cultures grown for 24 h at 25 °C without sodium selenite (**A**, **C**) and with 128 mM sodium selenite (**B**, **D**, **E**, **F**). GRU33 cell measures are presented in **C**, **D**, **E** and **F** (red). Nanoparticles found in the sodium selenite treatment measures are indicated in **E** and **F** (green). Magnification of 5000× in **A**–**D** and 20,000× in **E** and **F**

**Fig. 7:**
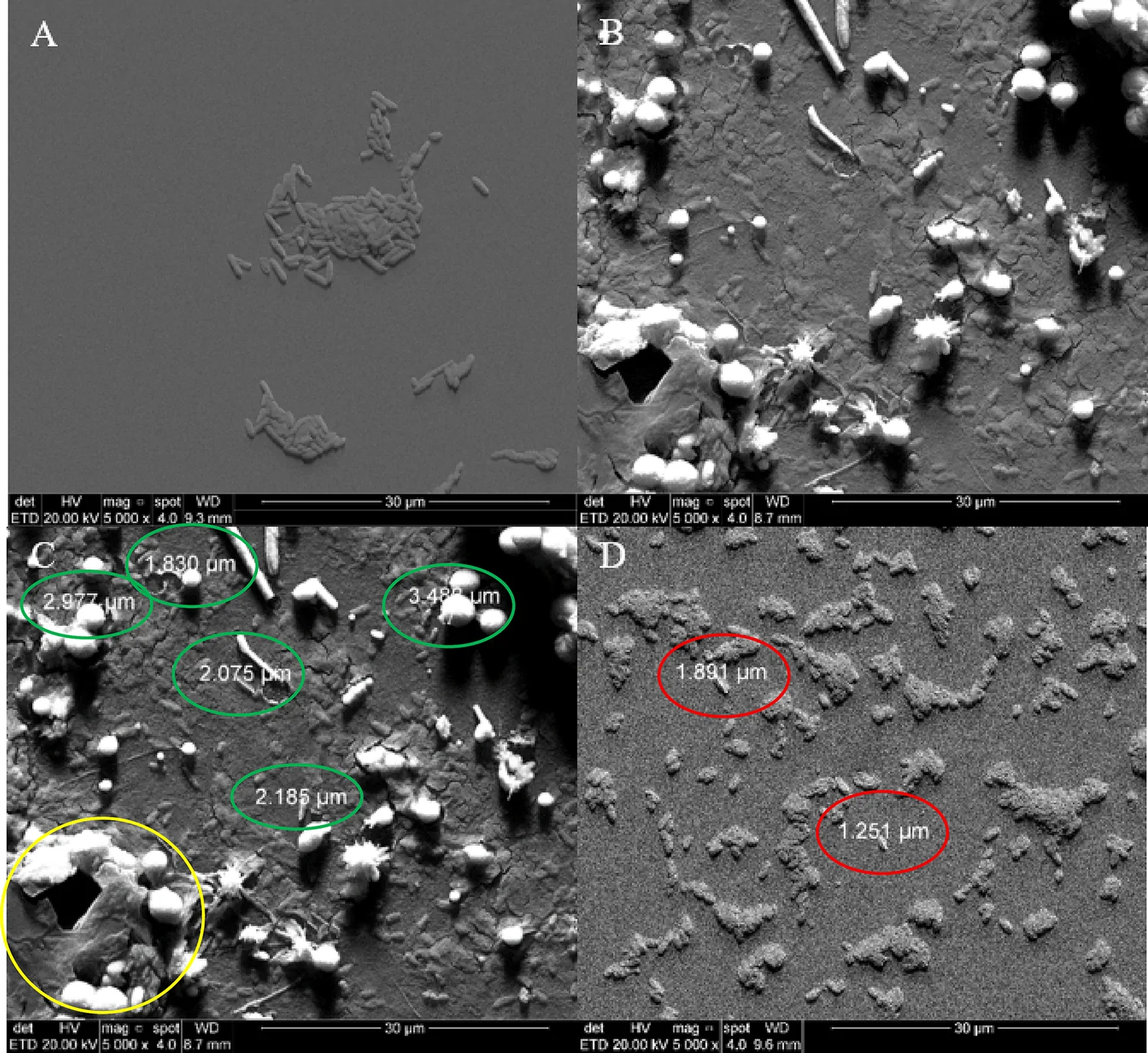
Scanning electron microscopy (SEM) analysis of CRG isolate MD330.11 (magnification of 5000×). MD330.11 cultures grown for 24 h at 25 °C without mercury (II) acetate (**A**, **D**) and treated with 0.06 mercury (II) acetate (**B**, **C**). Measures of particles (green) and MD330.11 cell size (red) are presented in **C** and **D**, respectively. An area presenting putative EPS with potential mercury particles is yellow-circled

**Fig. 8:**
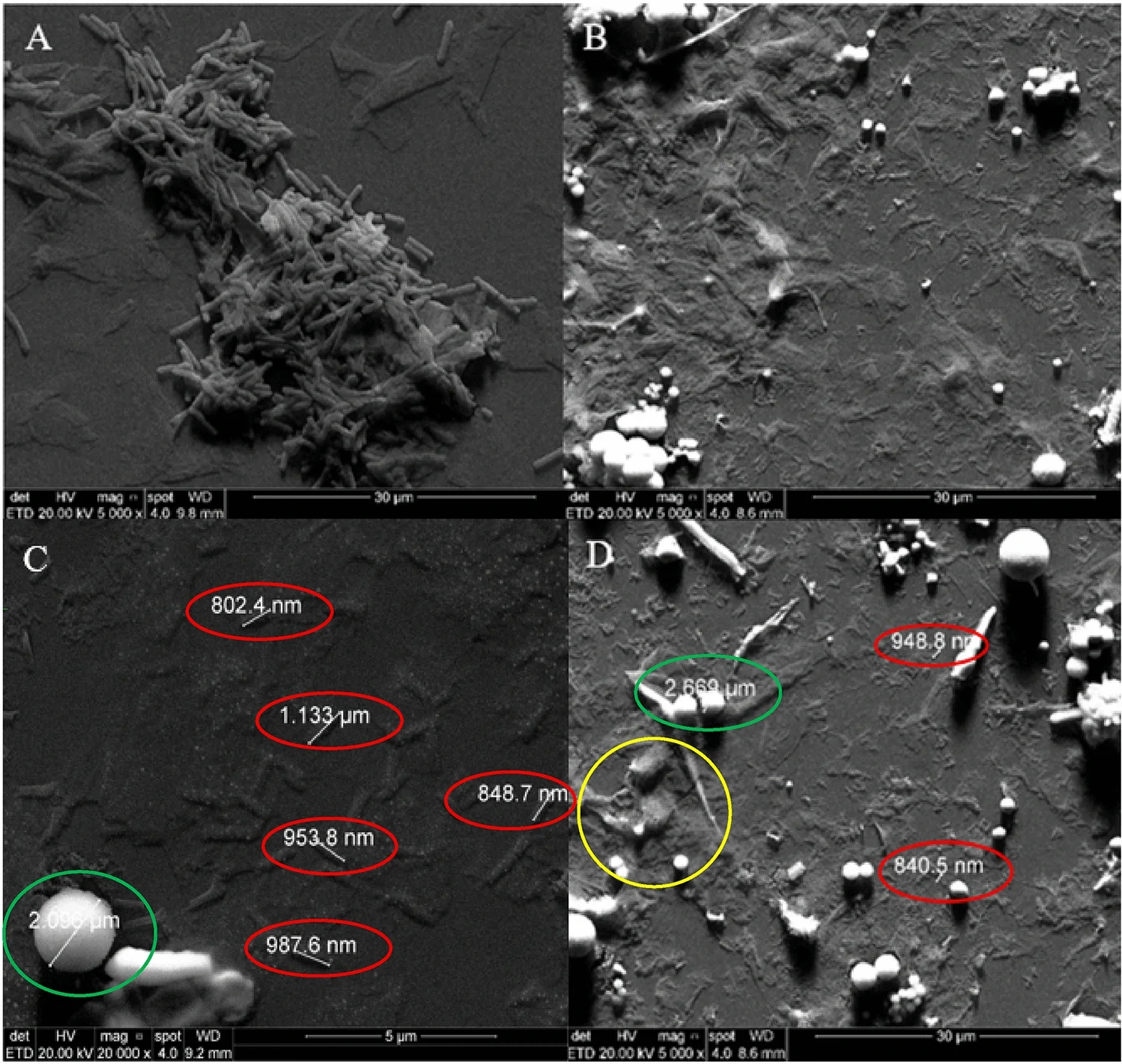
Scanning electron microscopy (SEM) analysis of CRG isolate MET16 (magnification of 5000×). MET16 cultures grown for 24 h, at 25 °C without mercury (II) acetate (**A**) and treated with 0.06 mercury (II) acetate (**B**, **C**, **D**). Measures of particles (green) and MET16 cell size (red) are presented in **C** and **D**, and MET16 cell size in **D** (red). An area presenting putative EPS with particles is yellow-circled

The SEM analysis aimed to obtain images of isolates from GAS (VB5 and GRU33) and RGC (MD330.11 and MET16) that presented high MIC values for the metals tested, to verify whether their cells presented morphological modifications when treated with sodium selenite (GAS) or mercury (II) acetate (RGC), as previously reported (De et al. 2008; Jia et al. 2014; Nancharaiah and Lens 2015; Wadhwani et al. 2016; Tugarova et al. 2020; Joshi et al. 2021). Elemental composition was assessed by energy dispersive X-ray spectroscopy (EDS), to investigate the potential presence of selenium nanoparticles (SeNPs) in selenium-treated GAS bacteria (Figs. 5, 6) and to characterize the mineral particles observed in mercury-treated RGC isolates (Figs. 7, 8).

SEM images from GAS isolates corroborated the biofilm data, with no mature biofilm observed for VB5 and GRU33 in either control or 128 mM sodium selenite treatments (Figs. 5, 6). Spherical extracellular nanoparticles (145.7–332.3 nm diameter) were observed exclusively in sodium selenite-treated cultures of both isolates, and EDS analysis confirmed a selenium-specific peak in these nanoparticles, consistent with their expected diameter for selenium nanoparticles (Supplementary Fig. S2). Secondary peaks (zinc, gold, titanium) were attributed to sample handling. A glass coverslip control showed no selenium signal. Bacterial cell morphology was also clearly altered under 128 mM sodium selenite treatment compared to controls (Figs. 5, 6).

SEM images from RGC isolates MD330.11 and MET16 similarly revealed spherical extracellular mineral particles under mercury (II) acetate treatment (1.830–3.488 µm diameter), absent in control cultures, along with fine needle and scalenohedral microstructures adhered to a matrix resembling bacterial EPS (Figs. 7, 8). EDS analysis confirmed a mercury-specific peak in these observed particles (Supplementary Fig. S3).

### Detection of Genes for mercury resistance (*merA*) and selenium metabolism (*selD*) 

Isolates that tolerated the highest concentrations of sodium selenite and mercury (II) acetate were tested for the presence of genes *selD* and *merA, respectively* . Five GAS isolates (VB5, GRU33, NSP5, MEL13 and CAP3) were analyzed for the presence of the *selD* gene, related to selenium incorporation in the pathway for selenoprotein synthesis, and eight RGC isolates (MD330.06, MD330.09, MD330.11, MD332.08, MET16, MR06, MR29 and MR38) for the *merA* gene, involved in mercury II reduction.

Among GAS isolates, only CAP3 presented the *selD* gene (approximately 500 bp) (Supplementary Table S1, Fig. S4). This isolate had a sodium selenite MIC of 128 mM, the second highest detected for this metal (Table 2), and had 24 h-biofilm induced by sodium selenite, mainly at 8 and 16 mM, with no biofilm formed under sodium selenite treatment in 48 h (Fig. 3).

For the *merA* gene, which encodes the mercury II reductase (MerA) enzyme, among the marine bacteria tested, all those encoding the *merA* presented the highest MIC value detected for mercury (II) acetate (0.125 mM). This is the case of isolate MR06 (*Bacillus megaterium*), which exhibited PCR results compatible to the presence of this gene (~ 400 bp) (Supplementary Fig. S4). Differently, the other two tested *Bacillus* (MR29 and MR28) had mercury (II) acetate MIC of 0.125 mM but did not amplify *merA.* This may indicate that the *merA* primers employed were not able to detect this gene in these isolates, or that these strains do not present this gene. Also, differently from MR29 and MR28, MR06 had its species identified as *B. megaterium*, which may have also contributed to these differences.

Furthermore, two *Pseudomonas* sp. (MD330.11 and MD332.08) also presented *merA* gene, however with a slight size difference of the amplified fragment (~ 600 bp) compared to the expected target fragment (431 bp) (Supplementary Fig. S4). As already mentioned, the *mer* operon was previously detected in *Pseudomonas* isolate MD330.06 through whole genome sequencing (NCBI BioProject PRJNA616366, Proença et al. 2022). Nonetheless, this gene could not be detected via PCR using general *merA* primers. The alignment search between the contig nucleotide sequence and the primers used indicated 100% identity of the forward primer with the c41 region between 14,915–14,932 bp. The reverse primer, however, had no similarity with the contig. This can be explained by the dissimilarity between the *merA* gene of *Pseudomonas* MD330.6 and the *Citrobacter freundii* reference, as an alignment between the two revealed only 91% identity. The target region of the reverse primer is particularly divergent, with only 63% identity. The resulting interspecific binding can also explain the amplification of larger fragments in the other *Pseudomonas* isolates, with a downstream binding region within the same gene resulting in an expected fragment of 674 bp, as we observed.

### Data compilation

Among all tested bacteria we observed a total of 10 isolates (6 from GAS and 4 from RGC) that presented results that can be highlighted. Table 4 compiles the maximum concentrations of metals these isolates resisted, their biofilm formation ability, the detection of genes involved in selenium incorporation and mercury reduction, as well as the occurrence of putative EPS and nano- or microparticles of selenium or mercury, respectively (observed by SEM images).

**Table 4:** Compilation of results of isolates from the Guarani Aquifer System and Rio Grande Cone that presented unique properties across our analyses

Env	Isolate	Maximum MIC (mM)						Biofilm		Genes	SEM
		Fe	Al	Se	Hg	Cu	Ni	24 h	48 h		
Guarani Aquifer System	*Pseudomonas* sp. CAP2	++	++	+				***	***	NT	NT
	*Bacillus* sp. CAV19	++	++	+				**	*	NT	NT
	***Bacillus* sp. VB5**	++	++	++				*		ND	Se nanoparticles
	***Lysinibacillus* sp. GRU33**	+	+	++				***		ND	Se nanoparticles
	*Unsup. Lysinibacillus* MEL13	+	+	+				**	**	ND	NT
	**Unidentified CAP3**	++	++	+				***		*selD*	NT
Rio Grande Cone	*Pseudomonas* sp. MD332.08				++	+	++	**		*merA*	NT
	***Pseudomonas* sp. MD330.11**				++	+	+	**	*	*merA*	EPS, Hg particles
	*Bacillus* sp. MR06				++	+	++	*		*merA*	NT
	*Paenibacillus* sp. MET16				++	+	+	*	*	NT	EPS, Hg particles

The isolates that had the highest MICs for each tested metal are indicated as “++”, while those with other high MICs values are marked as “+”. The ability of biofilm formation is noted as *** (strong producer), ** (moderate producer), or * (weak producer). PCR results for genes involved in selenium and mercury metabolism, and observations from SEM images are also informed. The four isolates with relevant combinations of properties are indicated in bold letters

*NT* not tested, *ND* not detected

Among GAS isolates, *Lysinibacillus* GRU33 and *Bacillus* VB5 were the only bacteria that presented two relevant features: the highest MIC value of sodium selenite and the presence of Se nanoparticles. Moreover, VB5 presented the highest MIC values also for iron (II) sulfate and aluminum sulfate, indicating a combination of relevant properties regarding tolerance to these heavy metals. Nevertheless, both these isolates were able to form biofilms only in 24 h under sodium selenite treatment. *Bacillus* CAV19 and *Pseudomonas* CAP2 also presented the highest MIC values for iron (II) sulfate and aluminum sulfate but formed stable biofilms along 24 and 48 h under all concentrations of sodium selenite tested. Additionally, CAP2 was the only isolate that presented mainly strong biofilms along all sodium selenite treatments. Even without *selD* gene detected, the combination of the high MIC values for iron (II) sulfate and aluminum sulfate with the property to form strong and stable biofilms when exposed to selenium, distinguishes this *Pseudomonas* isolate. Finaly, the unidentified isolate CAP3 also presented interesting results, with the highest MIC values for iron (II) sulfate and aluminum sulfate, and as the only isolate with *selD* gene detected. Interestingly, like VB5 and GRU33, this isolate did not form biofilms in 48 h sodium selenite treatments.

Among the selected marine isolates, the only *Bacillus* (MR06) and the two *Pseudomonas* (MD330.11 and MD332.08) reached the highest mercury (II) acetate MIC, and two of them (MR06 and MD332.8) presented this result also for nickel (II) chloride. Moreover, these three isolates also had the *merA* gene detected. Another apparent tolerance strategy, based on putative EPS production (that may capture microparticles of mercury), was observed in SEM images of the *Pseudomonas* MR330.11 and *Paenibacillus* MET16.

## Discussion

Heavy metals, especially at high levels, can accumulate in metal-tolerant microorganisms, interfere with their biochemical pathways and metabolic activities, in addition to interacting with cellular components (membranes, proteins and nucleic acids) through covalent and ionic bonds (Wang et al. 1997; Moreira and Moreira 2004; Hunt and Ward 2015). Some microbial populations can be significantly reduced, but the resistant and tolerant ones may propagate and eventually carry these elements through the food web (Hunt and Ward 2015). In this context, monitoring the emergence of metal-resistant strains in microbial communities, and the occurrence of genes that confer such resistance along water bodies, is of fundamental importance (Bengtsson-Palme and Larsson 2015; Berglund 2015; Gillard et al. 2019).

In 2018, hydrogeochemical and geophysical analysis were conducted on samples collected from 25 different locations in the GAS, most of which consisted of wells in the region of Candelária, Rio Grande do Sul, Brazil. Physical–chemical analysis of waters samples of this reservoir detected that iron (II) sulfate and selenium were found in excessive levels and above Maximum Residue Levels (MRLs) allowed throughout the sample area (average values of 6.5 mg/L for iron (II) sulfate and 0.015 mg/L for selenium), while aluminum sulfate showed medium to high levels (average of 0.160 mg/L), which varied between the different sampled wells (Moreno et al. 2021). According to the Consolidation Ordinance No. 396/2008 of the Brazilian National Environmental Council (Conselho Nacional de Meio Ambiente—CONAMA 2008), for human consumption, MRLs of iron, selenium and aluminum sulfate are 0.3 mg/L, 0.01 mg/L and 0.5 mg/L, respectively. The metals’ concentrations used to test our GAS isolates were above the detected levels of these metals in GAS samples by Moreno et al. (2021), with the maximum concentration used corresponding to 431.68 mg/L for aluminum sulfate, 853.44 mg/L for iron and 6906.8 mg/L for selenium. Although the original environmental conditions were not designed in the assays, the tolerance observed in GAS bacteria to such high concentrations is consistent with, and may reflect, a history of exposure to these heavy metals in this water reservoir. One isolate (VB5) from the region of Candelária proved to resist the highest concentrations tested of sodium selenite, aluminum sulfate, and iron (II) sulfate. Moreover, selenium concentration of 0.018–0.032 mg/L—above the allowed MRL (CONAMA 2008)—were detected in GAS samples specifically from Faxinal do Soturno municipality (Soares 2019), which is the region of the isolate GRU33 that also tolerated the highest concentration tested of selenium. The elevated concentration of selenium in this GAS site and high tolerance of the isolates to this metal is suggestive. However, our results are from one single isolate per site and on water-quality data collected independently of our sampling (Soares 2019; Moreno et al. 2021). Thus these observations should be interpreted as a hypothesis rather than direct evidence of local selection. Selenium is an essential micronutrient for organisms in general (Mangiapane et al. 2014), and in natural environments it is available in different organic and inorganic forms. In water it is usually dissolved either as selenite or selenate, whether in rocks and sediments it’s commonly found as metal selenide mineral. Microorganisms that can transform this element play a critical role in the selenium cycle, either by reduction or oxidation mechanisms (Pepper et al. 2014; Nancharaiah and Lens 2015). Both selenite and selenate are highly soluble and toxic forms of selenium that can be reduced by bacteria to a non-toxic insoluble form of elemental selenium (Se0) under anaerobic conditions (Garbisu et al. 1996; Hunter and Kanter 2009).

Some GAS isolates on which sodium selenite presented low MIC values seemed to counteract this result of “sensitivity” to sodium selenite when exposed to high concentrations of this metal during biofilm formation. Biofilm is an effective strategy for microbial survival and persistence under stress—like the exposure to antimicrobials and heavy metals—increasing their resistance to such stressful conditions compared to their planktonic counterparts (Muller et al. 2007; Dash et al. 2017). So, biofilm structure may be associated with a protective mechanism that allows bacteria to survive and persist in environments with high metal concentrations, especially when they are sensitive in planktonic condition (Muller et al. 2007; Mah and O'Toole 2001; Prakash et al. 2003; Teitzel and Parsek 2003). In this context, subinhibitory heavy metal concentrations are reported to induce biofilm formation, mainly in sensitive bacteria (Wu et al. 2015; Giovanella et al. 2017). In 24 h, our GAS isolates were all able to form biofilms in control and sodium selenite-treated conditions, independently of their selenium MIC. Thereafter, most isolates decreased their biofilm intensity in 48 h but still forming them under most sodium selenite concentrations. Nevertheless, *Pseudomonas* CAP2, which presented a low sodium selenite MIC compared to most isolates, maintained a strong biofilm pattern even at the highest concentrations of sodium selenite tested. Differently*, Lysinibacillus* GRU33 and *Bacillus* VB5, which are taxonomically close, exhibited the highest sodium selenite MIC and no biofilm formation in 48 h. This is consistent with the idea of these isolates rely on alternative, biofilm-independent strategies to tolerate high selenite concentrations, although these strategies were not directly investigated here. The results obtained for these (and other) GAS isolates suggest they may act as biocatalysts for the reduction of selenite, corroborating the results from previous studies with bacteria from the order Bacillales (Jia et al. 2011). When submitted to selenite, most GAS isolates exhibited orange-red colored colonies in MIC tests, as also did their selenite-treated biofilms. This indicates the presence of SeNPS, according to Nancharaiah and Lens (2015) and Wadhwani et al. (2016), which were later detected in SEM images of VB5 and GRU33 isolates. The SeNPS are reported as a bacterial product resulting from the detoxification of selenite (Wadhwani et al. 2016; Tugarova and Kamnev 2017). The synthesis of these nanoparticles can occur extracellularly, intracellularly, or membrane-bound, with a size variance of 24–500 nm (Garbisu et al. 1996; Oremland et al 2004; Dhanjal and Cameotra 2010; Wang et al. 2011; Dobias and Suvorova 2011; Zhang et al. 2011; Dwivedi et al. 2013), which is compatible to the particles' sizes we detected in MEV images. Another previous study also reported the metallic selenides-producing bacterium *Bacillus cereus* CC-1 as able to form SeNPs, indicating either multiple reduction mechanisms or the existence of specific SeNPs efflux pump in this strain. Several putative genes responsible for heavy metal resistance, salt resistance and selenate reduction were found in the genomic sequence of the strain. Among these, the authors detected the gene cluster narG-narH-narJ-narI, encoding the complete respiratory nitrate reductase, which is a putative selenate and selenite reductase, mainly involved in anaerobic selenite reduction (Che et al. 2019). Moreover, selenite reduction was already reported for strains of family *Pseudomonadaceae* (Hunter and Kanter 2009; Gonzales-Gil et al. 2016; Tendenedzai et al. 2020).

Alternatively, some bacteria can reduce selenate by incorporating it into organic compounds such as selenoproteins (Stolz et al. 2006; Lenz and Lens 2009; Nancharaiah and Lens 2015). The presence of *selD* gene can characterize the potential ability of selenium incorporation in proteins in the form of selenomethionine (SeMet) and selenocystein (Sec) aminoacids (Low and Berry 1996; Romero et al. 2005; Zhang et al. 2006; Estrada et al. 2020). All these processes are important and related to the conservation of metabolic energy for microorganisms and bioremediation of polluted environments (Knight et al. 2002; Stolz et al. 2006; Lenz and Lens 2009; Williams et al. 2013). Among GAS isolates the *selD* gene was detected only in CAP3 (unidentified), suggesting that this bacterium carries the genetic marker for selenoprotein production, which may represent a protective mechanism against selenium toxicity, although this was not functionally tested. Even with this difference from the Bacillales VB5 and GRU33 isolates, CAP3 also presented no biofilm formation in 48 h. All these results reinforce that biofilms are more intensively promoted under metal stress mainly for bacteria that are metal-sensitive in planktonic condition (Muller et al. 2007; Wu et al. 2015; Giovanella et al. 2017), whereas highly tolerant isolates (independently of their tolerance mechanism) seem not to need biofilms to survive under long term metal exposure. Given their high selenite/selenate tolerance, VB5, GRU33 and CAP3 may represent promising candidates for further investigation as bioremediation strains for selenite/selenate-contaminated sites, though functional bioremediation assays would be needed to confirm this potential.

The heavy metals used to test GAS isolates can be found in natural environments in organic and inorganic state (Bigham et al. 2002; Boyd 2012; Sposito 2020). Iron is commonly found in groundwater due to the dissolution of the ore by carbon dioxide, which forms soluble ferrous carbonate in water, (the most common element in this environment) (Freeze and Cherry 1979; CETESB 2009), and in ferrous state forms soluble compounds (Parron et al. 2011). Aluminum sulfate can be found as soluble salt, colloids, or insoluble forms. It occurs in ores, rocks, and clays (Freeze and Cherry 1979; CETESB 2009). However, the occurrence of any sample having at least one metal with levels above the MRLs (CONAMA 2008), can be considered as an alert of a significant impact. These elements rarely occur naturally in high concentrations in groundwater (CETESB 2009; Freeze and Cherry 1979). It is important to emphasize that this is a deep reservoir that holds huge volumes of water, presenting small number of outcrop zones and little natural metal recharge (Araújo et al. 1999; Teramoto et al. 2020). Nevertheless, the contamination of these environments has been increasing in the last decades, as a result from human activities. The lack of adequate protection of these valuable water bodies against human discharges and leachate, associated with the poor planning of wells’ drilling, allows the direct entry of polluted water or shallow groundwater, which leads to a physical–chemical imbalance of these deep reservoirs (Foster et al. 2009). GAS sites from which the bacterial strains were isolated are in a region of the Brazilian state Rio Grande do Sul that traditionally presents intensive agricultural activities, according to Secretary of Planning, Budget and Management in the Socioeconomic Atlas of Rio Grande do Sul (2019). Thus, bacteria from these GAS sites have been subjected to the continuous presence of elevated levels of different classes of contaminants, including heavy metals (Soares 2019; Moreno et al. 2021). A previous study with these same GAS isolates also revealed that several among them presented tolerance to herbicides and/or resistance to antibiotics (Silva et al. 2025). This is the case of CAP2, CAV19, RF3121, MEL13 and CAE1. Our present data are consistent with the presence of strains exhibiting increased tolerance to at least some heavy metals in this aquatic system, although our cross-sectional design does not allow us to establish whether this reflects an ongoing selective process. The association of increasing loads with the occurrence of metal-resistant bacteria has been previously reported (Bhardwaj et al. 2018). Moreover, bacterial isolates from pristine waters of a hot spring in South Africa were evaluated in relation to their antibiotic resistance and heavy metal tolerance. A total of 37.5% of the isolates were resistant to one or two antibiotics, while the remaining 22% exhibited complete sensitivity (Jardine et al. 2019). The co-resistance of metals and antimicrobials in bacteria from aquatic ecosystems is an issue of increasing concern worldwide (Baker-Austin et al. 2006; Pal et al. 2017; Squadrone 2020).

Different from the GAS isolates, among which some bacteria presented a high level of tolerance to the heavy metals tested, the bacteria from the RGC showed the lowest MIC values, especially of mercury (II) acetate. Mercury is a highly toxic element, with the ability to invade cytoplasm and deactivate enzymes (Seigneur et al. 1997). All the marine isolates we tested were sensitive to this metal in planktonic condition. Barros et al. (2014) also reported marine *E. coli* isolates from the beaches of São Luís (MA, Brazil), located in a highly polluted site, which were tolerant to all the heavy metals tested (copper, nickel, cadmium), except for mercury. Nevertheless, our *Pseudomonas* MD332.08 can be highlighted, since it exhibited the highest MIC values for the three heavy metals tested. Moreover, due to the dilution effect, concentrations of heavy metals occurring in oceanic environments are usually much lower than those observed in continental water bodies (Zhuang and Gao 2014), even when compared to GAS, one of the biggest aquifer systems in the world. The MRLs allowed for Brazilian saline waters are 0.005 mg/L for copper, 0.0002 mg/L for mercury and 0.025 mg/L for nickel, according to Consolidation Ordinance No. 357/2005 of CONAMA (CONAMA, 2005). The maximum concentrations used in this work to expose isolates were far above these (corresponding to 1016.64 mg/L of copper, 25.07 mg/L of mercury and 939.04 mg/L of nickel).

Heavy metal particles can be transported by water, adsorbed on sediment particles, and thus accumulated in different compartments of the marine environment (Duleba et al. 2018). Bacterial communities from deep marine sediments can present cellular mechanisms related to the ability to concentrate metals (Fonti et al. 2015), which points to a potential capacity for metal accumulation and bioremediation in their natural environment that merits further investigation. Metal accumulation was registered effectively bound to putative EPS produced by bacterial cells as well (Jroundi et al. 2019). In this study, production of EPS-like structures was observed in SEM images from two marine isolates under mercury (II) acetate treatments. EPS have adsorptive and adhesive properties, containing high molecular weight compounds charged with functional groups, which confers polyanionic nature (Wilen et al. 2003). These polymers accumulate nutrients from the environment and participate in the degradation of particles, biocorrosion and bioleaching of minerals, and biosorption of heavy metals (Beech et al. 2005; Gadd 2010; Jia et al. 2011). Furthermore, they can also contain enzymes that support detoxification of heavy metals by transformation and precipitation of these elements in the polymeric mass (Jia et al. 2011; Panwichian et al. 2011). In this context, evidence of mercury trapping in biofilm-EPS was reported for a mercury-resistant marine *Bacillus cereus* strain (Dash et al. 2017). Moreover, nanoparticles of inorganic mercury were previously detected with diameters range 70–120 nm in *Lysinibacillus sphericus* surface (Vega-Páez et al. 2019). In the present study, microparticles of needle, scalenohedral and spherical forms with a high mercury content were also found in the SEM images, which seemed to be adhered to the “EPS surface” of marine isolates. However, the particles we detected were much higher in size (than those previously reported), ranging from 1.8 to 3.4 µm. Nevertheless, peculiarities of bacterial cell's surface and specific transporters related to *mer* operon can interfere in the sizes of possible particles to be adhered to bacterial surface (Schaefer et al. 2011; Zheng et al. 2012; Gilmour et al. 2013). So, our SEM and EDS data are compatible with these bacterial isolates trapping small mercury particles, possibly as a protective mechanism, as previously suggested (Oyetibo et al. 2016), although this interpretation remains speculative in the absence of direct functional evidence.

The PCR analysis performed with our marine isolates indicated that *Pseudomonas* MD332.8 and *Bacillus megaterium* MR06 [both exhibiting the highest mercury (II) acetate MIC] carry the *merA* gene. This gene codes for the mercury II reductase (MerA), which transforms toxic mercury II into a relatively non-toxic volatile form of this metal. Thus, *merA* is the most surveyed gene in mercury bioremediation studies that involves the detection of *mer* operon (Barkay et al. 2003). *Pseudomonas* sp., *Bacillus* sp. and *Pseudoalteromonas* sp. from deep-sea were previously described as morphologically and genetically adapted to the exposure of heavy metals, and due to activity of *merA* gene, they were able to reduce >80% inorganic mercury (Joshi et al. 2021). Nevertheless, MR06 did not form any biofilm and MD332.08 formed only in 24 h. MD332.08 exhibited a pattern similar to the *Bacillus cereus* BW-201B strain, which was able to tolerate 50 ppm of mercury and formed biofilm in the presence of this metal, with mercury binding to EPS reported within 24 h of incubation in that study; after this period, *mer*-mediated volatilization of mercury reportedly reached its optimum, with biofilm disintegration coinciding with internalization of EPS-bound mercury (Dash et al. 2017). We did not directly test mercury volatilization or EPS-bound mercury internalization in our isolates, so this comparison is offered as a plausible parallel rather than a confirmed mechanism in MD332.08.

Differently, *Pseudomonas* MD330.11, besides presenting *merA* gene, also showed biofilms that tolerated the highest mercury (II) acetate concentration tested, and visual evidence (in SEM images) of producing some EPS when treated with this metal, which is consistent with a higher biofilm-mediated response to mercury stress compared to other RGC isolates. A similar result was already reported by Giovanella et al (2017), which also evidenced the increase in biofilm formation by an isolate of *Pseudomonas* sp. in the presence of mercury (II) acetate. These characteristics may render stability and resistance to survive in a mercury contaminated environment (Dash et al. 2017; Joshi et al. 2021) and may also indicate the ability of biosorption of heavy metals, like mercury, as previously reported in several strains of *L. sphaericus*, *B. thuringiensis* and *P. aeruginosa* (Hassen et al. 1998; Vega-Páez et al. 2019). Additionally, both these features—the presence of *merA* gene and the extracellular absorption of mercury—point to a potential of *Pseudomonas* MD330.11 for bioremediation applications, by either reducing or immobilizing this heavy metal in the environment, warranting further functional characterization (De et al. 2006; Francois et al. 2012; Joshi et al. 2021).

Furthermore, in the present study, the products of *merA* amplification presented different sizes in relation to the target fragment, which may suggest polymorphisms among our isolates regarding this gene (Felske et al. 2003; Oregaard and Sørensen 2007; Rasmussen et al. 2008). Additionally, as mentioned, the *Pseudomonas* isolate MD330.6 had this gene annotated in its sequenced genome (NCBI BioProject PRJNA616366, Proença et al. 2022), but did not have this gene amplified in our experiments, which is another evidence of *merA* polymorphism (at least regarding the fragment we used). Moreover, it is also possible that different variants of this gene that remain unidentified may occur among bacterial taxa (Møller et al. 2014).

The *mer* operon is a complex gene structure, since over evolutionary time new functionalities have been selected to increase the efficiency of mercury II detoxification and uptake. A great diversity and distribution of genes encoding the MerA enzyme were found and recorded in complete genomes of environmental isolates (Christakis et al. 2021). About 1–10% of microorganisms cultivated from different environments present the *mer* system (Barkay 1987), which indicates the ubiquity of mercury II in nature and the selective pressure this metal imposes on the microbiota for detoxifying it. Therefore, the wide distribution of nearly identical MerA proteins among different groups of bacteria may be associated with a combination of factors such as highly conserved proteins, selective pressure, and horizontal transfer (Osborn et al. 1997).

Additionally, studies have reported the frequent association between *mer* operon and antimicrobial resistance (Baker-Austin et al. 2006; Mathema et al. 2011; Dash et al. 2013; Cao et al. 2024). The presence of the *merA* gene was related to resistance to a variety of antibiotics and other toxic heavy metals of a marine *B. thuringiensis* (Dash et al. 2013), and in antibiotic-resistant *Pseudomonas aeruginosa*, in which *merA* was associated to resistance to four different antimicrobials (Cao et al. 2024). As already mentioned above (for other heavy metals), this is a point of high concern regarding resistance to mercury, especially in aquatic systems, as previously highlighted (Baker-Austin et al. 2006; Pal et al. 2017; Squadrone 2020). The presence of bacteria with multiple resistance profiles can cause direct changes in the ecology of communities of marine microorganisms. Also, many resistant microbial strains from natural habitats may belong to species potentially pathogenic to the human population (Barros et al. 2014).

Some limitations of this study should be acknowledged. First, the number of isolates per genus and per sampling site was small, which limits general interpretations regarding the geographic and taxonomic associations proposed here. In this context, some apparent patterns (e.g., the co-occurrence of high selenite tolerance and local contamination reports) should also be interpreted with caution. Second, MIC and biofilm assays were performed in vitro, under standardized laboratory conditions with a single metal at a time and constant exposure, which does not fully reproduce the fluctuating, multi-stressor conditions of the natural environment, or the presence of competing/interacting microbial communities. Third, comparisons of percentages of isolates across MIC categories were descriptive, not supported by formal statistical testing, given the sample size. Finally, the environmental contamination data used to contextualize GAS results (Moreno et al. 2021; Soares 2019) were obtained independently of the present isolates’ sampling, so the link between reported metal levels and the phenotypes observed here is inferential rather than direct. Nevertheless, our data are still relevant, as they reveal important phenotypic traits of bacteria from poorly explored deep aquatic environments.

The contamination of water bodies from industrial activities, agriculture, fish farming and mining has been evidenced as an emerging global problem and, consequently, the microbiota of those environments is severely impacted (Kennish 2002; Häder et al. 2020). So, inadequate planning of areas that suffer high anthropogenic impact can generate continuous efflux of heavy metals and other damaging xenobiotics into the environment, with considerable effects (Korropati and Megendale, 2005; Deycard et al. 2014). Brazil stands out for its agriculture, livestock and industries. Therefore, it is a great target for investigations about tolerance to heavy metals in microorganisms from aquatic habitats, especially those that are poorly investigated for this purpose. In this context, the results raised in this study reinforce the importance of monitoring the environmental impacts of anthropic origin to reconcile human development with sustainability, in line with the one health principle.

## Conclusions

This study reports relevant information regarding metal tolerance of bacteria from two Brazilian deep aquatic ecosystems, revealing some phenotypic patterns and detecting candidate genes potentially involved in metal stress in these microorganisms. GAS isolates exhibited higher tolerance to iron (II) sulfate, aluminum sulfate, and sodium selenite, with some strains forming strong biofilms under sodium selenite stress, producing particles consistent with elemental selenium nanoparticles, and showing morphological adaptations. Notably, some isolates displayed high sodium selenite MICs, the presence of orange-red pigmentation and extracellular nanoparticles consistent with selenite reduction. However, it is important to note that the underlying mechanism was not directly analyzed via direct measurements of selenite reduction rates or selenium speciation. In contrast, RGC isolates were more sensitive to mercury in planktonic conditions, displayed weak biofilm formation, and showed extracellular mercury-containing particles adhered to EPS-like matrices, a pattern compatible with mercury sequestration and suggestive of alternative tolerance strategies. Moreover, the detection of *selD* and *merA* genes in GAS and RGC isolates, respectively, indicates the presence of candidate genetic markers potentially associated with metal detoxification pathways, though their expression levels or functionality were not experimentally tested in this study. These findings highlight the adaptive potential of microbial communities inhabiting deep aquatic systems, particularly in response to anthropogenic and natural metal contamination. The observed phenotypic and genetic diversity in heavy metal tolerance underscores the ecological relevance of these bacteria in maintaining ecosystem function under chemical stress but also raises concerns regarding tolerant traits that can act against other biocides, like antibiotics. Continued exploration of these scarcely investigated environments, combined with functional validation of the candidate mechanisms identified here, may reveal novel microbial mechanisms of tolerance and contribute to sustainable strategies for managing metal pollution in aquatic ecosystems.

## Supplementary Information

Below is the link to the electronic supplementary material.

Supplementary file1 (PDF 460 KB)

## Data Availability

No datasets were generated or analysed during the current study.
