# Supplementary material for: Tolerance to heavy metals of bacteria from Brazilian deep aquatic environments
Source: Arch Microbiol. 2026 Oct 10;209(1):4. doi: 10.1007/s00203-026-05226-5 (PMC13653815; doi:10.1007/s00203-026-05226-5)
Supplement: Supplementary file 1 [file 203_2026_5226_MOESM1_ESM.pdf]

Supplementary material

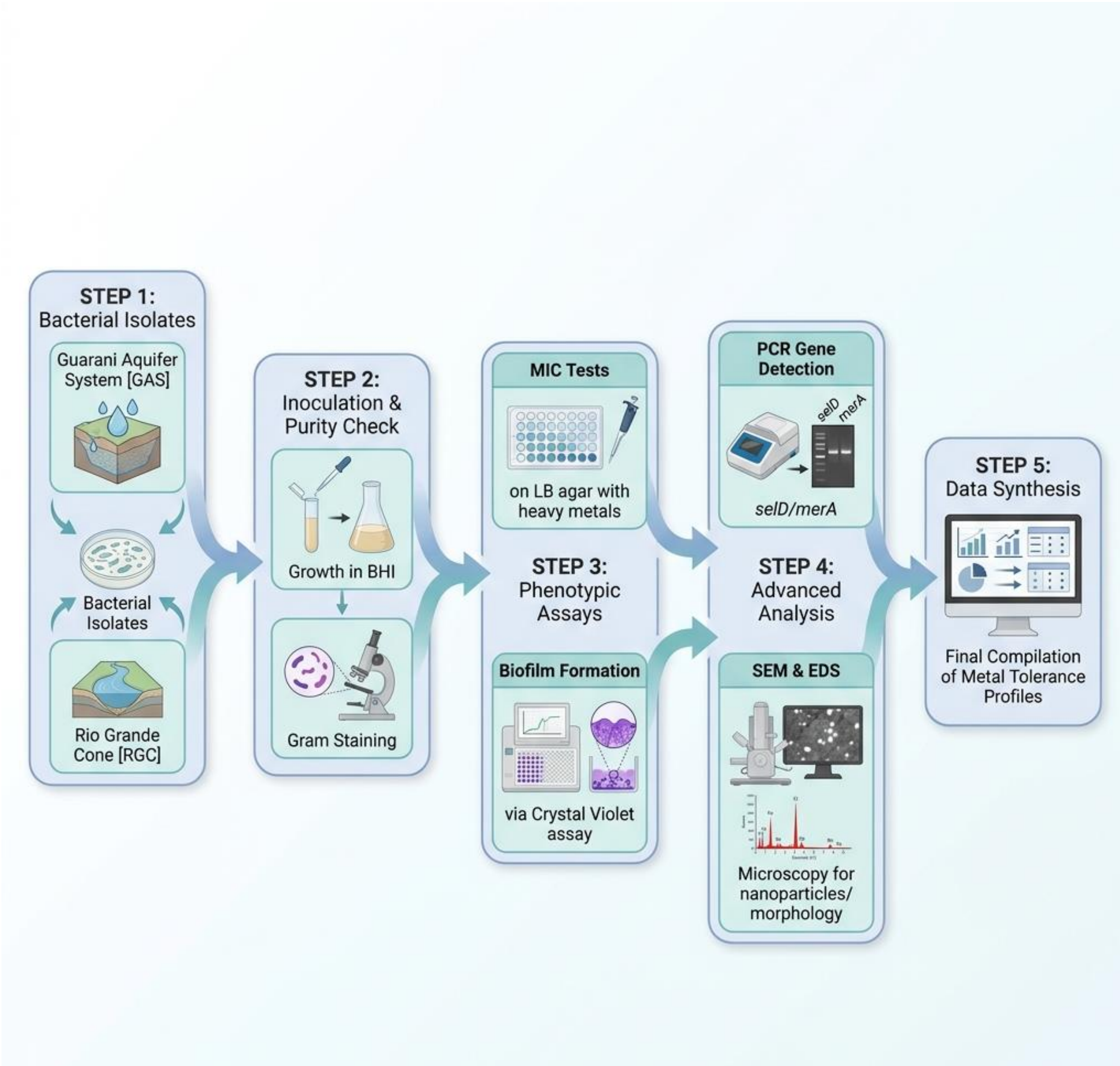

Figure S1. Schematic workflow summarizing isolates' origin, minimal inhibitory concentration (MIC) testing, biofilm assays, scanning electron microscopy (SEM), and molecular analyses. Created in Google Notebook LM.

A

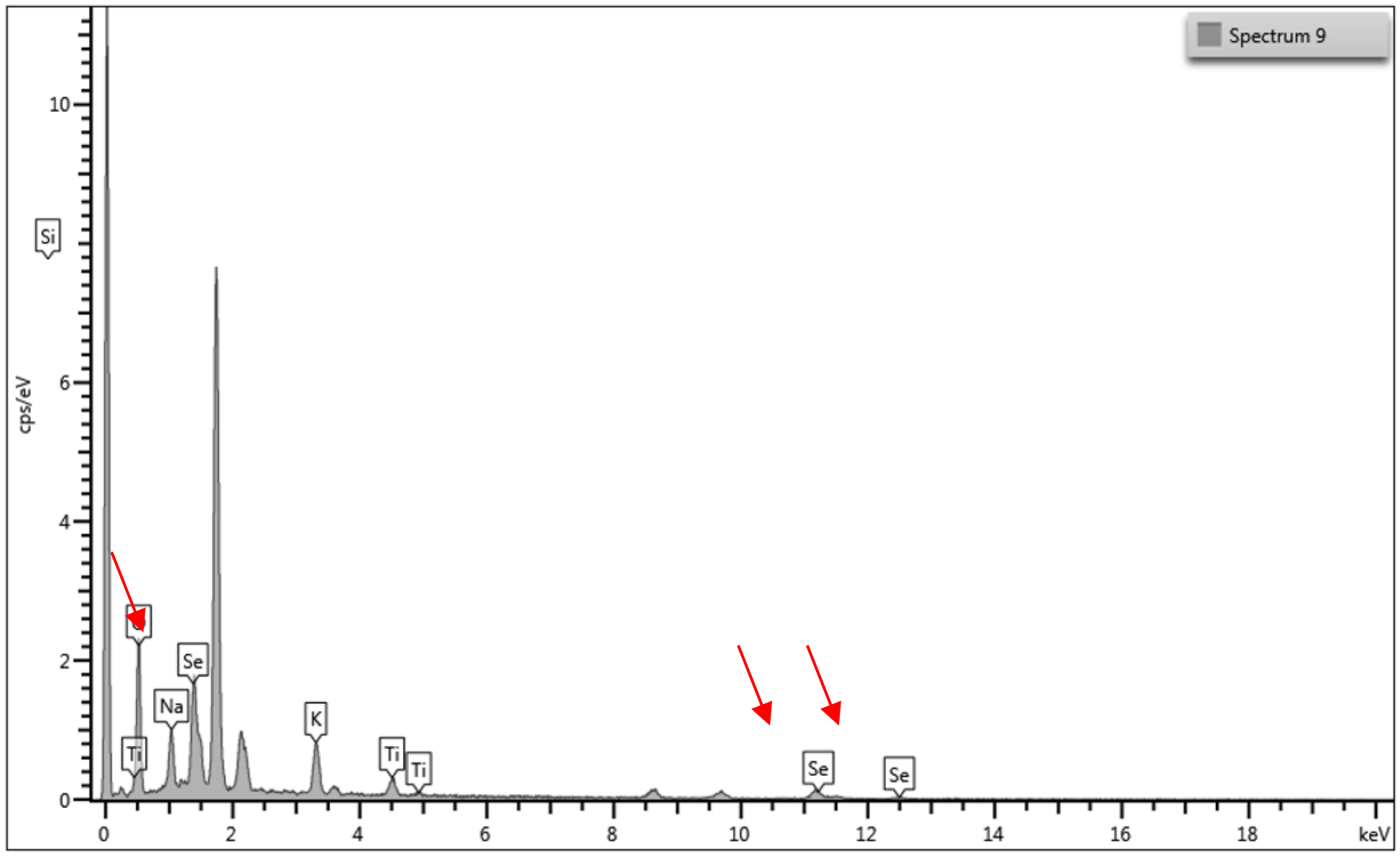

B

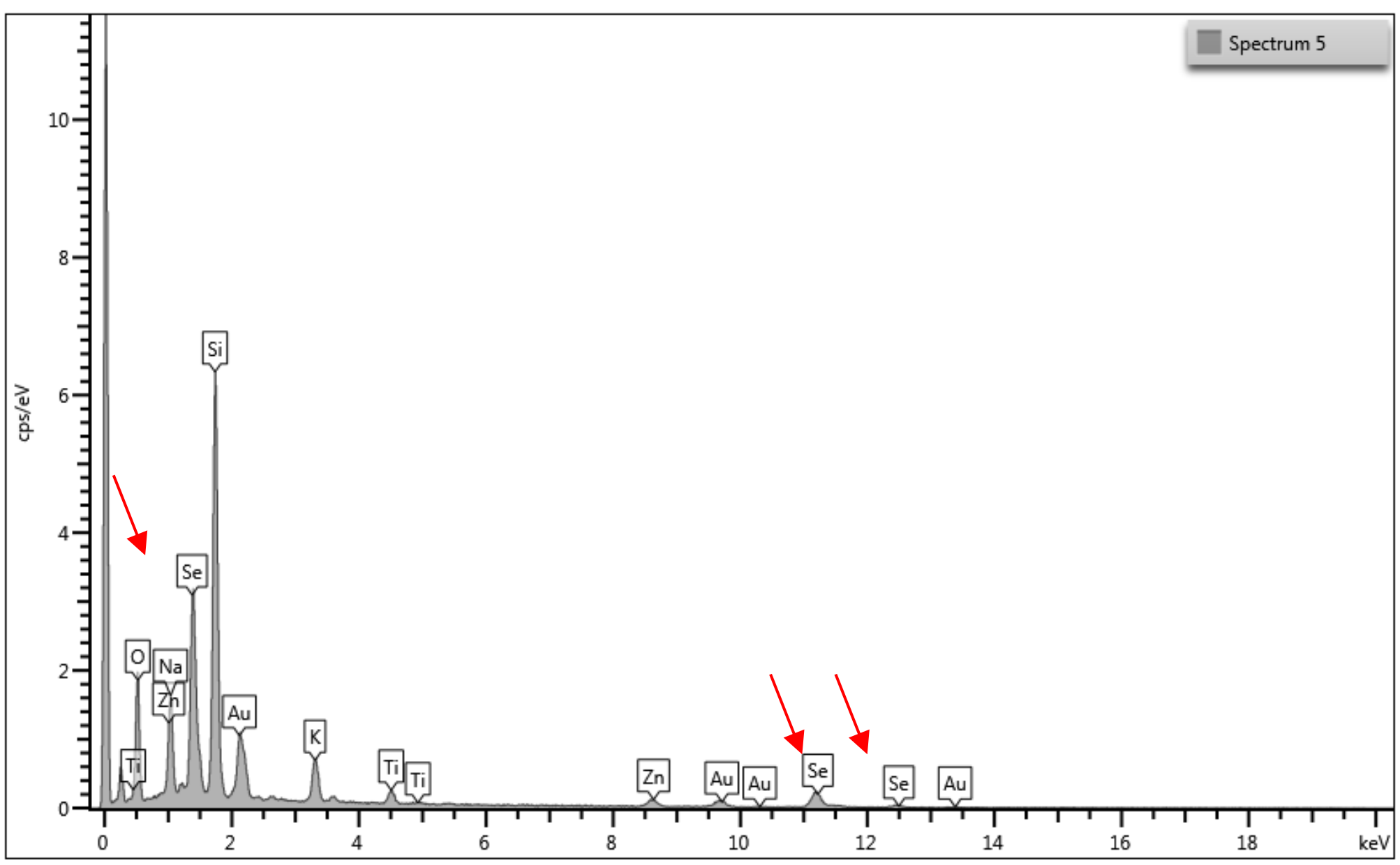

Figure S2. Results from Scanning Electronic Microscopy with EDS analysis of the nanoparticles present in VB5 (A) and GRU33 (B) cultures treated with 128 mM of sodium selenite.

A

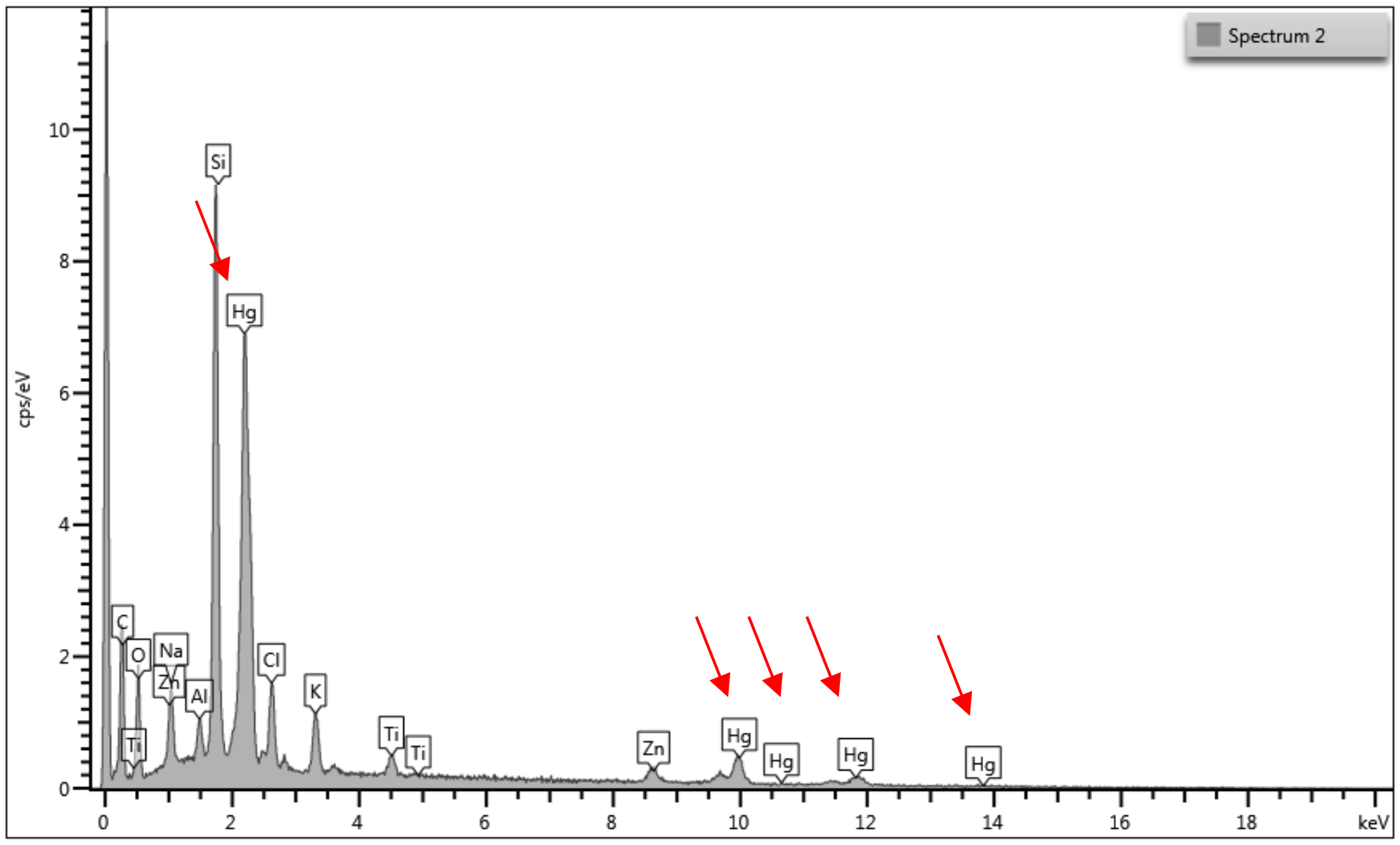

B

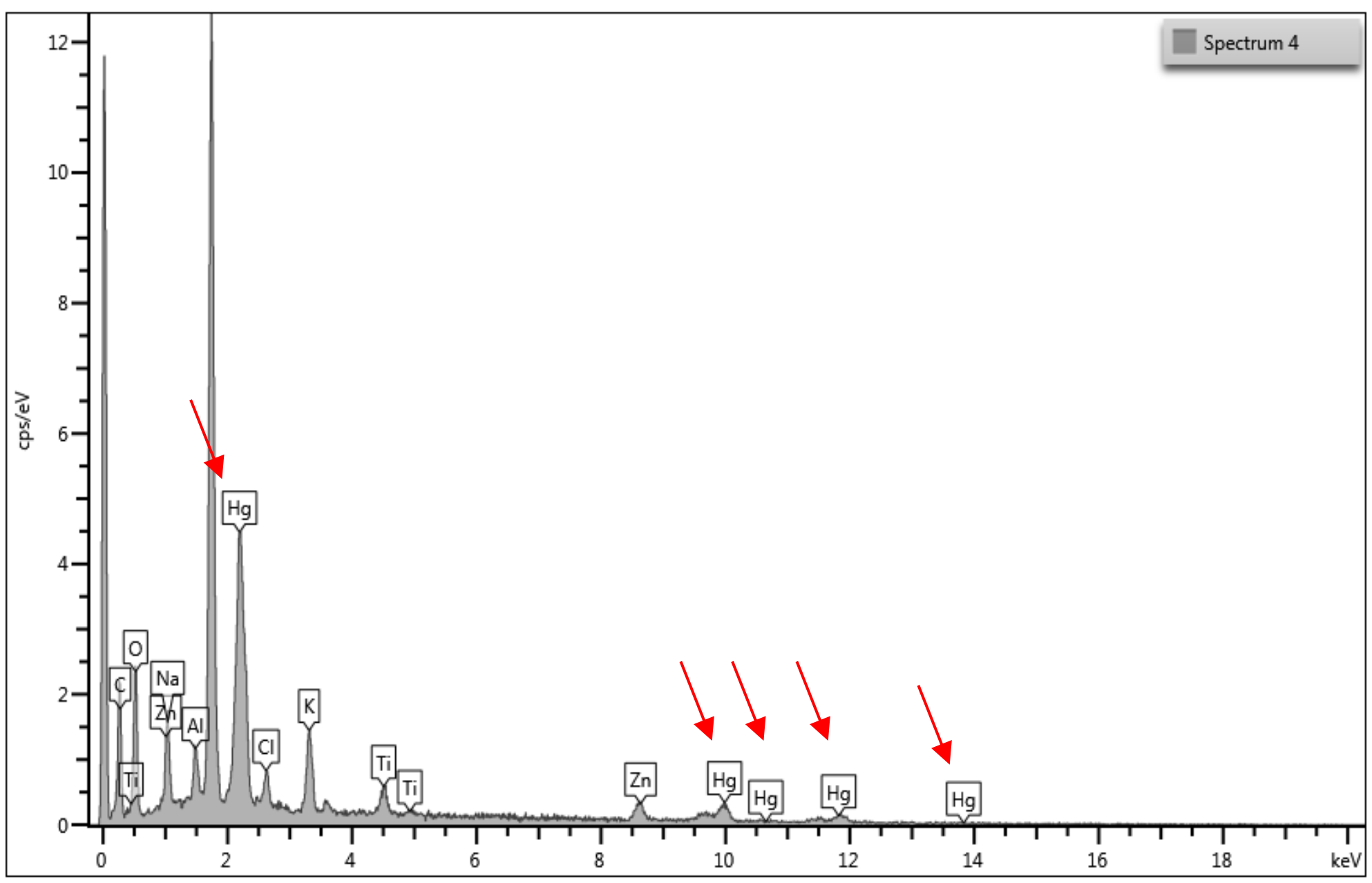

Figure S3. Results from Scanning Electronic Microscopy with EDS analysis of the particles present in MD330.11 (A) and MET16 (B) cultures treated with 0.06 mM of mercury acetate. The high silicon peak is probably from the coverslip used to fix the samples. The potassium peak was due to the presence of this element in the buffer used for the preparation of the samples.

Table S1. Isolates from the Guarani Aquifer System (GAS) and Rio Grande Cone (RGC) that were investigated for the presence of selenium or mercury resistant genes, respectively. The isolates that had positive PCR results are indicated in grey for the respective genes. Values of minimum inhibitory concentrations (MIC) of selenium (Se) and mercury (Hg) obtained for each isolate (from Tables 2 and 3) are also indicated.

**GAS Isolates**	**Taxonomic identification**	**Se MIC (mM)**	***selD***	**RGC Isolates**	**Taxonomic identification**	**Hg MIC (mM)**	***merA***
VB5	*Bacillus*	256		MD330.6	*Pseudomonas* sp.	0.06	
GRU33	*Lysinibacillus*	256		MD330.9	*Pseudomonas* sp.	0.06	
MEL13	Unsup. *Lysinibacillus*	128		MD330.11	*Pseudomonas* sp.	0.125	
NSP5	Unidentified	128		MD332.8	*Pseudomonas* sp.	0.125	
CAP3	Unidentified	128		MET16	*Paenibacillus sp.*	0.125	
				MR06	*Bacillus megaterium*	0.125	
				MR29	*Bacillus* sp.	0.125	
				MR38	*Bacillus* sp.	0.125	

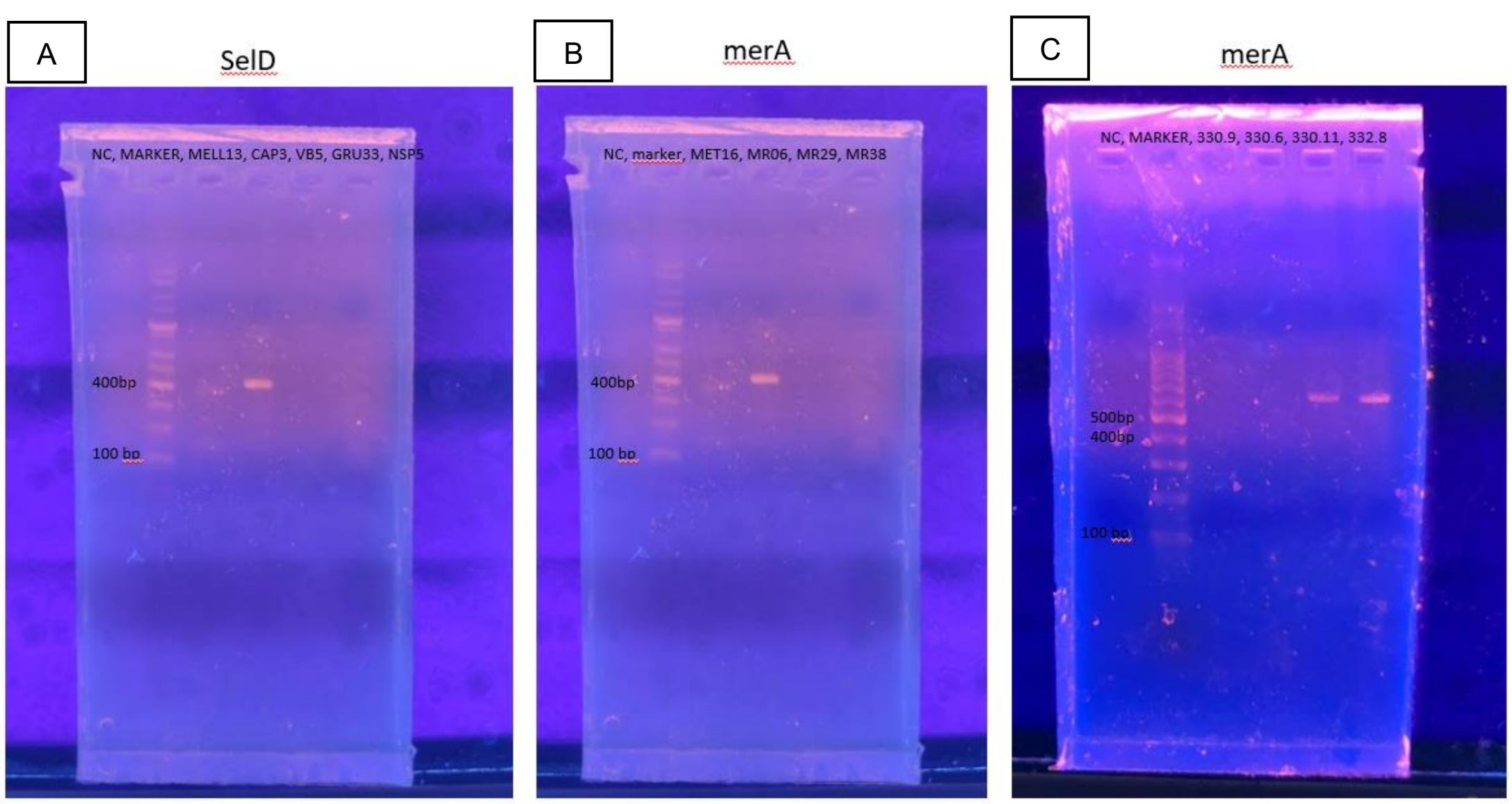

Figure S4. Conventional PCR results (in agarose gels) of metal-resistance genes amplification. Results of *selD* gene from GAS isolates MELL13, CAP3, VB5, GRU33 and NSP5, in which only CAP3 presented a positive amplification (A); Results of *merA* gene from RGC isolates MET16, MR29, MR38, MD330.9, MD330.6, MD330.11 and MD332.8, with positive amplifications for MR06 (B), MD330.11 and MD332.8 (C).
